# Excess adiposity and cancer: evaluating a preclinical-clinical obesity framework for risk stratification

**DOI:** 10.1016/j.eclinm.2025.103247

**Published:** 2025-05-12

**Authors:** Michael F. Leitzmann, Michael J. Stein, Hansjörg Baurecht, Heinz Freisling

**Affiliations:** a Department of Epidemiology and Preventive Medicine, University of Regensburg, Regensburg, Germany; b Nutrition and Metabolism Branch, International Agency for Research on Cancer (IARC/WHO), Lyon, France

**Keywords:** Clinical obesity, Cancer risk, Cohort study

## Abstract

**Background:**

Obesity is a known cancer risk factor, yet its classification by body mass index fails to capture organ dysfunction.

**Methods:**

In 459,342 UK Biobank participants enrolled between 2006 and 2010, we applied the Lancet Diabetes and Endocrinology Commission's definitions of preclinical (excess adiposity without organ dysfunction) and clinical obesity (with organ dysfunction) to prospectively assess associations with 28 cancers. Multivariable Cox regression estimated hazard ratios and 95% confidence intervals for each obesity classification and cancer type.

**Findings:**

During 11.6 years of follow-up, 47,060 incident cancer cases were identified. Preclinical obesity was positively associated with 11 cancer types across multiple organ systems, including cancers of the digestive (esophageal adenocarcinoma, gastric cardia, liver, biliary tract, pancreas, colorectum), reproductive (endometrium, postmenopausal breast, fatal prostate), urinary (kidney), and endocrine (thyroid) systems. Clinical obesity was positively associated with 12 cancers, showing stronger relations particularly for metabolically driven malignancies such as hepatocellular carcinoma and endometrial, colorectal, and pancreatic cancers. It was also positively associated with lung cancer. Conversely, preclinical and clinical obesity were inversely associated with non-fatal prostate cancer, suggesting a distinct underlying mechanism. We estimate that in the UK Biobank cohort, preclinical obesity accounted for 5.5% (4.7, 6.3) and clinical obesity for 4.3% (3.6, 4.9) of obesity-related cancer.

**Interpretation:**

The link between preclinical obesity and increased cancer risk suggests that obesity-related carcinogenesis begins before clinically detectable abnormalities, highlighting the need for early risk identification. Stronger associations with clinical obesity, particularly in metabolically driven cancers, reinforce the role of organ dysfunction in exacerbating carcinogenesis, emphasizing medical monitoring and intervention.

**Funding:**

Funding for IIG_FULL_2021_027 was obtained from World Cancer Research Fund (WCRF UK), as part of the World Cancer Research Fund International grant programme. This study was supported by the French National Cancer Institute (l’Institut National du Cancer, INCA_16824) and the 10.13039/501100001659German Research Foundation (BA 5459/2-1).

**Research in context**

**Evidence before this study**

Previous research has primarily relied on BMI to assess adiposity in relation to cancer risk, despite its inability to capture fat distribution, body composition, or the metabolic activity of adipose tissue. Major reports from organizations such as the International Agency for Research on Cancer (IARC, a specialized cancer research agency of the World Health Organization) and the World Cancer Research Fund/American Institute of Cancer Research (WCRF/AICR) have provided valuable insights using BMI; however, they do not address obesity's metabolic heterogeneity. Recently, the Lancet Diabetes & Endocrinology Commission introduced a revised framework that differentiates preclinical obesity - excess adiposity without organ dysfunction - from clinical obesity, where excess adiposity coexists with dysfunction in physiological systems.

**Added value of this study**

This study is the first to prospectively examine cancer risk using the revised obesity framework by directly comparing preclinical obesity, clinical obesity, and the traditional BMI-based definition. By examining 28 malignancies in the UK Biobank cohort, our analysis reveals that classifying obesity by the presence or absence of organ dysfunction uncovers nuanced obesity-cancer associations. Specifically, preclinical obesity was linked to increased cancer risk even in the absence of detectable organ dysfunction, whereas clinical obesity generally conferred a greater risk, particularly for metabolically-driven cancers.

**Implications of all the available evidence**

Our results suggest that obesity-related carcinogenesis involves both excess adiposity and its metabolic consequences. Refining obesity classification by distinguishing preclinical from clinical obesity may improve risk stratification and support targeted prevention strategies.

## Introduction

Body mass index (BMI) is the most widely used metric for assessing body fatness and serves as a key risk factor in epidemiologic research and in guiding public health policies. While effective for population-level categorization, BMI does not account for fat distribution or body composition. Consequently, it fails to distinguish between metabolically active and inactive adipose tissue, limiting its validity in assessing obesity-related disease risk.^1^

To address these limitations, the Lancet Diabetes & Endocrinology Commission proposed a revised obesity framework.^2^ Moving beyond the traditional view of obesity as a homogeneous disease risk factor, the Commission distinguished between preclinical obesity, defined by excess adiposity (confirmed through direct fat measurement or a secondary anthropometric measure such as waist circumference alongside BMI, or assumed for BMI >40 kg/m^2^) without major organ dysfunction, and clinical obesity, characterized by excess adiposity plus organ dysfunction affecting physiological systems (i.e., neurological, respiratory, cardiovascular, metabolic, hepatic, renal, urinary, reproductive, lymphatic, and musculoskeletal) or causing substantial mobility limitations in daily activities. By incorporating organ function and mobility status into obesity assessment, this classification potentially enhances disease risk stratification. This framework also reinforces the distinction between obesity as a risk factor for disease and clinical obesity as a pathological condition in its own right, characterized by specific signs, symptoms, and physiological dysfunctions that define it as a chronic disease entity, warranting recognition and management independent of its associations with other outcomes.

Given these distinctions, understanding how different forms of obesity relate to cancer is particularly important. Mechanistically, obesity is strongly linked to cancer through pathways involving altered lipid metabolism, insulin resistance, chronic inflammation, and adipokine dysregulation.^3^ While obesity can foster a pro-tumorigenic environment, cancer initiation and progression require additional tissue-specific and molecular alterations, such as genomic instability and immune evasion.^4^ Thus, the etiologic pathways underlying cancer differ from those driving obesity.

Although BMI-based reports, including those from the International Agency for Research on Cancer (IARC)^5^ and the World Cancer Research Fund (WCRF),^6^ have provided key insights into obesity-related cancer risk, they may not have fully captured the nuances of obesity's effects on cancer. The Lancet Commission's obesity classification offers a more pathophysiologically relevant approach to assessing obesity, potentially overcoming some of BMI's limitations. However, its relevance to cancer risk remains unexamined. This study aims to fill that gap by prospectively investigating preclinical and clinical obesity in relation to total and site-specific cancer incidence in a large cohort.

## Methods

### Study population and data collection

The UK Biobank is a prospective cohort study that recruited 502,134 UK participants aged 40–69 years between 2006 and 2010. Data on sociodemographic factors, lifestyle behaviors, and phenotypic characteristics were collected through touchscreen questionnaires, interviews, physical assessments, and biological samples. Ethical approval was granted by the North West Multi-Centre Research Ethics Committee, and all participants provided written informed consent.^7^ For the present analysis, we excluded participants with missing anthropometric data (n = 3209), those with BMI <25 kg/m^2^ but a waist circumference >102 cm (men) or >88 cm (women) (n = 3251), and individuals with prevalent cancers at baseline (n = 36,332). The final analytical sample included 459,342 participants (Supplemental Figure S1).

### Anthropometric data and clinical obesity criteria

At baseline, trained clinical staff measured height using a Seca 202 stadiometer and weight using a Tanita BC-418 body composition analyzer. Waist circumference was measured at the narrowest part of the torso or the navel during exhalation.^8^ BMI was calculated as weight (kg) divided by height squared (m^2^).

We applied the diagnostic criteria for preclinical and clinical obesity as proposed by the Lancet Diabetes and Endocrinology Commission.^2^ We defined preclinical obesity as excess adiposity, operationalized as a BMI ≥25 kg/m^2^ plus a waist circumference >88 cm in women or >102 cm in men [per WHO (World Health Organization) guidelines^9^], or assumed for BMI >40 kg/m^2^, without signs or symptoms of organ dysfunction or obesity-related functional limitations. We defined clinical obesity as excess body fat meeting the same anthropometric criteria, but accompanied by signs of organ dysfunction and/or substantial limitations in daily activities due to obesity.

To identify obesity-induced organ dysfunction, we used a pre-specified list of ICD-10 diagnoses from hospital inpatient records up to baseline, supplemented by self-reported baseline indicators. These covered dysfunction across physiological systems specified by the Commission, including neurological (G93.2, H53.4), respiratory (G47.3, J96.1, R06.2), cardiovascular (I50, I48, I27, I26.9, I10), metabolic (E78), hepatic (K76.0, K74.0), renal (N18.1-5), urinary (N39.4), reproductive (N97.0, N91.3-5, E28.2, E29.1), and lymphatic (I89.0) systems. Musculoskeletal dysfunction was assessed using the UK Biobank question: “In the last month, have you experienced any of the following that interfered with your usual activities?”, specifically focusing on chronic, severe knee or hip pain. Mobility limitations due to obesity were identified using ICD-10 codes for falls (R29.6), impaired walking (R26.2), and need for personal care assistance (Z74.0), as well as self-reported data on shortness of breath, leg pain, or chest pain/discomfort while walking on level ground. These were based on the following UK Biobank questions: “Do you get short of breath walking with people of your own age on level ground?” (UKB field ID 4717); “Do you get pain when you walk at an ordinary pace on the level?” (ID 5485); and “Do you get this pain or discomfort when you walk at an ordinary pace on the level?” (ID 3606), the latter of which followed a question about experiencing chest pain or discomfort. These measures were selected to operationalize the Commission's framework as comprehensively as possible using available data. A summary of the mapping between diagnostic indicators and the Commission's domains is provided in Table 1.

**Table 1:** Alignment of clinical obesity diagnostic criteria with the **L**ancet **C**ommission framework.

Organ	Category	ICD-10 code/self-report source	Notes
CNS	Signs of raised intracranial pressure	G93.2, H53.4	Fully aligned
Upper airways	Apnoea/hypopnoea during sleep	G47.3	Fully aligned
Respiratory	Hypoventilation, breathlessness, or wheezing	J96.1, R06.2	Fully aligned
Cardiovascular	HFrEF and HFpEF	I50	Fully aligned
	Chronic/recurrent atrial fibrillation	I48	
	Pulmonary artery hypertension	I27	
	Recurrent DVT and/or pulmonary embolism	I26.9	
	Raised arterial blood pressure	I10	
Metabolism	Hyperglycaemia, high triglycerides, low HDL	E78	Fully aligned
Liver	NAFLD with hepatic fibrosis	K76.0, K74.0	Fully aligned
Renal	Microalbuminuria with reduced eGFR	N18.1–5	Fully aligned
Urinary	Recurrent/chronic urinary incontinence	N39.4	Fully aligned
Reproductive	Anovulation, oligomenorrhoea, PCOS, male hypogonadism	N97.0, N91.3, N91.4, N91.5, E28.2, E29.1	Fully aligned
Musculoskeletal	Chronic, severe knee or hip pain	Pain type(s) experienced in last month (UKB field ID: 6159)	Partial alignment; reliance on self-report noted as limitation
Lymphatic	Lower limb lymphoedema	I89.0	Fully aligned
Limitations in day-to-day activities		R29.6, R26.2, Z74.0; Shortness of breath walking on level ground (UKB field ID 4717) Leg pain when walking normally (UKB field ID 5485) Chest pain or discomfort walking normally (UKB field ID 3606)	Partial alignment; reliance on self-report noted as limitation

CNS: Central nervous system; DVT: Deep vein thrombosis; eGFR: Glomerular filtration rate; HDL: High density lipoprotein; HFpEF: Heart failure with preserved ejection fraction; HFrEF: Heart failure with reduced ejection fraction; ICD: International Classification of Diseases; NAFDL: Non-alcoholic fatty liver disease; PCOS: Polycystic ovary syndrome; UKB: UK Biobank.

### Cohort follow-up and cancer case ascertainment

Follow-up extended from the baseline questionnaire date until the first primary cancer diagnosis, loss to follow-up, death, or end of follow-up (December 2020 for England, November 2021 for Scotland, December 2016 for Wales),^10^ whichever came first. Vital status was determined through linkage to healthcare data databases and death registries.^11^ We identified first primary malignant cancers using ICD-10 and ICD-O-3 codes, considering multiple cancer diagnoses on the same day (n = 1524 instances) as a single case, selected at random. To ensure robust statistical power, we included only cancers with ≥100 cases, resulting in 28 cancer types in the final analysis. This included fatal prostate cancer, which we assessed separately from non-fatal cases (Table 2).

**Table 2:** Classification of cancer endpoints.

Cancer	ICD-10 code	ICD-O-3 code
Head and neck (incl. oral, pharynx, larynx)	C0–C14 and C30–C33, C37, C38	–
Oesophagus (adenocarcinoma)	C15	8140, 8141, 8143–8145, 8190–8231, 8260–8263, 8310, 8401, 8480–8490, 8550–8551, 8570–8574, 8576
Oesophagus (squamous cell carcinoma)	C15	8050–8078, 8083, 8084
Stomach (cardia)	C16.0	8140–8145, 8147, 8210, 8211, 8214, 8220, 8221, 8230, 8231, 8255, 8260–8263, 8310, 8480, 8481, 8490, 8510, 8560, 8562, 8570–8576
Stomach (non-cardia)	C16.1–C16.6	8140–8145, 8147, 8210, 8211, 8214, 8220, 8221, 8230, 8231, 8255, 8260–8263, 8310, 8480, 8481, 8490, 8510, 8560, 8562, 8570–8576
Colorectum	C18–C20	–
Liver (hepatocellular carcinoma)	C22.0	–
Biliary tract	C22.1, C23, C24	–
Pancreas	C25	–
Lung	C34	–
Melanoma	C43	–
Breast (post-menopausal)	C50	Defined by self-reported menopausal status or ≥ 55 years
Breast (pre-menopausal)	C50	Defined by self-reported menopausal status or < 55 years
Cervix	C53	–
Corpus uteri	C54.0, C54.1, C54.2, C54.3, C54.9, C55	–
Ovary	C56	
Prostate (non-fatal)	C61 without death during follow-up	–
Prostate (fatal)	C61 with C61 as cause of death	
Kidney (renal cell carcinoma)	C64	8050, 8140, 8260, 8270, 8280–8312, 8316–8320, 8340–8344
Bladder	C67	–
Glioma	C71, C72	9380–9384, 9391–9460
Thyroid	C73	–
Hodgkins lymphoma	C81	–
Non-Hodgkins lymphoma	C82 to C85 (excluding C83.3)	–
Diffuse large B-cell lymphoma	C83.3	–
Multiple myeloma	C90	–
Lymphoid leukemia	C91	
Myeloid leukemia	C92	–

ICD: International Classification of Diseases.

### Covariates

Potential confounders were identified by a panel of four senior researchers with expertise in cancer epidemiology and obesity research (MFL, MJS, HB, HF), who reached consensus through iterative discussion informed by prior literature and causal inference principles. Models were stratified by sex, study center, and age (5-year increments). Adjustments were made for height (continuous), education (college/university degree; higher national diploma, A-level, or equivalent; general certificate of secondary education, O-level; none), socioeconomic status (Townsend index, continuous), smoking status (never, former, current), alcohol consumption (never, former, current), diet (healthy diet score, continuous), physical activity (metabolic equivalent of task (MET)-hours/week, quartiles), sedentary behavior (0–3 h per day, 4–5 h per day, 6–7 h per day, 8–24 h per day), and previous cancer screening (bowel, breast, prostate cancer screening). For analyses involving female-specific cancers, additional adjustments were made for age at menarche (<12 years, 12–14 years, >14 years), number of live births (0, 1, 2, 3, ≥4), oral contraceptive use (binary), age at natural menopausal (continuous), hysterectomy status (binary), and hormone replacement therapy use (binary). Missing data were handled using missing indicator variables to retain the maximum sample size.^12^

### Statistical analysis

Cox proportional hazards regression, with age as the underlying time metric^13^ was used to estimate hazard ratios (HRs) and 95% confidence intervals (CIs) for obesity classifications in relation to cancer incidence. Obesity was categorized according to the Lancet classification^2^ into three groups: no obesity, preclinical obesity, and clinical obesity, as previously defined. For comparison, adiposity was also classified according to the WHO's BMI-based definition as normal weight (BMI <25 kg/m^2^), overweight (BMI 25–29.9 kg/m^2^), and obesity (BMI ≥30 kg/m^2^).^14^ Pairwise comparisons between obesity categories were conducted using the Wald test. To account for multiple testing, the Benjamini-Hochberg correction (false discovery rate)^15^ was applied to all analyses, including the pairwise comparisons.

The proportional hazards assumption was assessed using Schoenfeld residuals^16^ and visual inspection, with no violations detected. Potential effect modification of the obesity-cancer relation by sex, age, smoking, and, among women, hormone replacement therapy was assessed through formal interaction tests using multiplicative terms and stratified analyses within these subgroups.

Various sensitivity analyses were conducted to assess the robustness of the primary analysis. These included: excluding the first two years of follow-up and underweight individuals to assess whether pre-existing undiagnosed conditions or low body weight might be consequences of underlying cancer rather than causes; adjusting for ethnicity (White/Mixed/Asian/Other) and family history of cancer (lung, bowel, breast, or prostate cancer in the mother and/or father) to address residual confounding; applying multiple imputation by chained equations (five datasets, five iterations each) to evaluate whether the use of missing indicator variables in the primary analysis materially affected the results; and restricting analyses to ICD-based indicators of organ dysfunction and mobility limitations to examine potential self-reporting bias.

In addition, the population attributable fraction (PAF) of UK Biobank cancers attributable to preclinical and clinical obesity was estimated using Levin's formula,^17^ assuming causal relationships. All p-values were derived from two-sided tests, with statistical significance set at an alpha level of 0.05. All data processing and statistical analyses were performed using R 4.4,^18^ with Cox regression implemented through the rms package.^19^

### Role of the funding source

Funding for IIG_FULL_2021_027 was obtained from World Cancer Research Fund (WCRF UK), as part of the World Cancer Research Fund International grant programme. This study was supported by the French National Cancer Institute (l’Institut National du Cancer, INCA_16824) and the German Research Foundation (BA 5459/2-1).

## Results

### Characteristics of the study population

We analyzed baseline characteristics of participants (n = 459,342) using the Lancet Commission classification. Sixty-seven percent had no obesity, 20% had preclinical obesity, and 13% had clinical obesity. In the no obesity group, participants were nearly evenly split between BMI <25 kg/m^2^ (48%) and 25–29.9 kg/m^2^ (47%), with 4% having a BMI ≥30 kg/m^2^ (Table 3). Most individuals with preclinical or clinical obesity had a BMI ≥30 kg/m^2^, more common in clinical obesity (70%) than preclinical obesity (61%), while a BMI of 25–29.9 kg/m^2^ was more frequent in preclinical obesity (39%) than clinical obesity (30%). Thus, clinical obesity was associated with a slightly higher average BMI (32.9 vs. 31.6 kg/m^2^), larger waist circumference, and greater subcutaneous and visceral adiposity than preclinical obesity. These anthropometric patterns describe average body size and fat distribution across groups, but do not reflect the criteria used to define clinical obesity, which additionally required documented organ dysfunction or mobility limitation.

**Table 3:** Age-standardized baseline characteristics of UK Biobank participants (N = 459,342) by the Lancet Diabetes and Endocrinology Commission classification of preclinical and clinical obesity.^a^

Characteristic	No obesity	Preclinical obesity	Clinical obesity
**N**	307,361	91,317	60,664
**Age** (years)	55.8 (8.2)	56.7 (7.8)	58.3 (7.7)
**WHO classification of adiposity**			
Normal weight	48.3%	0%	0%
Overweight	47.3%	38.8%	29.6%
Obesity	4.4%	61.2%	70.4%
**Sex**			
Women	51.1%	55.8%	58.4%
Men	48.9%	44.2%	41.6%
**Body mass index** (kg/m^2^)	25.1 (2.9)	31.6 (4.1)	32.9 (4.9)
**Waist circumference** (cm)	84.1 (9.9)	102.5 (10.1)	104.9 (11.5)
**Subcutaneous adipose tissue** (L)^b^	5.8 (6.3)	9.7 (10.4)	10.3 (13.9)
Missing	26.2%	28.6%	29.4%
**Visceral adipose tissue** (L)^b^	3.3 (5.5)	5.5 (8.0)	5.8 (10.2)
Missing	26.2%	28.5%	29.2%
**Height** (cm)	168.5 (9.4)	168.9 (9.9)	167.6 (9.8)
**Education**^c^			
Highest	35.0%	27.8%	20.8%
Intermediate	22.7%	23.6%	23.1%
Lowest	25.2%	27.0%	26.3%
Other	15.3%	19.4%	27.5%
Missing	1.7%	2.2%	2.2%
**Townsend index**	−1.5 (3.1)	−1.2 (3.2)	−0.6 (3.4)
Missing	0.1%	0.1%	0.1%
**Healthy diet score**	3.6 (1.4)	3.4 (1.4)	3.3 (1.4)
Missing	0.1%	0.2%	0.1%
**Physical activity** (MET-hours/week)^d^	47.3 (45.3)	39.34 (41.7)	36.6 (41.7)
Missing	21.2%	25.2%	30.2%
**Sedentary behavior** (hours/day)	4.3 (2.5)	4.9 (2.7)	5.4 (2.9)
Missing	597 (0.2%)	308 (0.3%)	159 (0.3%)
**Smoking status**			
Never	56.4%	51.7%	46.3%
Former	32.9%	38.4%	42.0%
Current	10.3%	9.3%	11.0%
Missing	0.4%	0.6%	0.7%
**Alcohol drinking status**			
Never	3.8%	4.5%	6.6%
Former	3.1%	3.3%	6.1%
Current	92.9%	91.9%	87.0%
Missing	0.2%	0.3%	0.3%
**Cancer screening**^e^			
Previous	66.5%	68.4%	75.7%
Never	33.4%	31.5%	24.2%
Missing	0.1%	0.2%	0.1%
**Age at menarche** (years)^f^	12.6 (4.1)	12.3 (4.0)	12.3 (3.9)
Missing	1.6%	1.9%	1.8%
**Number of live births**^f^	1.8 (1.7)	1.9 (1.7)	2.0 (1.7)
Missing	0.1%	0.2%	0.1%
**Oral contraceptive use**^f^			
No	18.0%	20.4%	22.2%
Yes	81.7%	79.0%	77.3%
Missing	0.4%	0.6%	0.5%
**Age at menopause** (years)^f^	49.9 (9.3)	50.1 (9.0)	49.3 (10.3)
Missing	6.4%	6.3%	7.1%
**Menopausal hormone use**^f^			
Never	59.5%	59.0%	49.8%
Ever	40.1%	40.3%	49.5%
Missing	0.4%	0.6%	0.7%

a Age standardization was done by direct standardization to the age distribution of the cohort at baseline. Values are mean (standard deviation) for continuous variables and proportions for categorical variables.

b Subcutaneous and visceral adipose tissue were measured during the imaging visit (starting 2014); the relative numbers of missing values refer to participants who attended the imaging visit.

c Highest education: college or university; intermediate: A/AS, NVQ/HND/HNC or equivalent, other qualifications; lowest: O/GCSEs, CSEs or equivalent.

d MET: metabolic equivalent of task.

e Bowel, breast, or prostate cancer screening.

f Among women.

Compared to participants with no obesity, both preclinical and clinical obesity were associated with lower education, poorer socioeconomic status, lower diet quality, less physical activity, and more sedentary behavior. Former smoking and past alcohol use were more common in both obesity groups, with current smoking more prevalent among those with clinical obesity. However, both obesity groups also exhibited healthier behaviors, such as lower current alcohol use and higher cancer screening rates. Women were more likely than men to have preclinical (21% vs. 19%) and clinical obesity (15% vs. 12%), whereas men were more likely than women to fall into the no obesity category (69% vs. 64%). Among women, clinical obesity was linked to higher rates of early menarche and menopausal hormone use, factors positively related to female cancers, but also to higher parity, oral contraceptive use, hysterectomy, and slightly younger age at menopause, which are associated with lower risk.

### Cancer risk associations

During 11.6 median years of follow-up (5,092,404 person-years), we identified 47,060 cancer cases (Supplemental Table S1). Preclinical obesity, compared to no obesity, was positively associated with 13 cancers after adjusting for other risk factors (Table 4). The strongest associations were observed for endometrial cancer and hepatocellular carcinoma (both HRs >1.5), followed by esophageal adenocarcinoma, cancers of the kidney, gastric cardia, thyroid, biliary tract, prostate (fatal), colorectum, pancreas, breast (postmenopausal), bladder; and diffuse large B-cell lymphoma (all HRs <1.5). Preclinical obesity was more strongly related to obesity-related cancers combined (HR = 1.29, 95% CI = 1.25–1.34) than to all cancers combined (HR = 1.09, 95% CI = 1.06–1.12). It showed no relation to lung cancer and was inversely associated with esophageal squamous cell carcinoma and non-fatal prostate cancer. After accounting for multiple testing, relations with bladder cancer and diffuse large B-cell lymphoma lost statistical significance (Table 4).

**Table 4:** Hazard ratios and 95% confidence intervals for preclinical, clinical obesity, and BMI-based obesity in relation to cancer types.

Cancer type	Obesity group	Cases	HR (95% CI)	FDR-adjusted p-value
Biliary tract	No obesity	277	1.00	
	Preclinical obesity	121	1.29 (1.04, 1.61)	0.0418
	Clinical obesity	120	1.69 (1.34, 2.11)	<0.0001
	BMI-based obesity	175	1.39 (1.09, 1.76)	0.0141
Bladder	No obesity	651	1.00	
	Preclinical obesity	245	1.18 (1.01, 1.37)	0.0637
	Clinical obesity	187	1.23 (1.04, 1.45)	0.0388
	BMI-based obesity	335	1.32 (1.11, 1.56)	0.0044
Breast (post-menopausal)	No obesity	3709	1.00	
	Preclinical obesity	1573	1.19 (1.12, 1.26)	<0.0001
	Clinical obesity	1115	1.18 (1.10, 1.27)	<0.0001
	BMI-based obesity	1747	1.32 (1.23, 1.41)	<0.0001
Breast (pre-menopausal)	No obesity	1690	1.00	
	Preclinical obesity	470	1.00 (0.90, 1.11)	0.9845
	Clinical obesity	270	1.00 (0.88, 1.15)	0.9845
	BMI-based obesity	518	0.94 (0.84, 1.05)	0.3970
Cervix	No obesity	77	1.00	
	Preclinical obesity	23	0.94 (0.58, 1.52)	0.9085
	Clinical obesity	16	1.00 (0.57, 1.76)	0.9993
	BMI-based obesity	28	1.07 (0.65, 1.76)	0.9117
Colorectum	No obesity	3457	1.00	
	Preclinical obesity	1348	1.23 (1.15, 1.31)	<0.0001
	Clinical obesity	856	1.12 (1.03, 1.21)	0.0120
	BMI-based obesity	1571	1.22 (1.13, 1.31)	<0.0001
Diffuse large B-cell lymphoma	No obesity	470	1.00	
	Preclinical obesity	177	1.20 (1.01, 1.43)	0.0788
	Clinical obesity	122	1.17 (0.95, 1.44)	0.2053
	BMI-based obesity	219	1.32 (1.08, 1.61)	0.0137
Endometrial	No obesity	622	1.00	
	Preclinical obesity	488	2.34 (2.07, 2.64)	<0.0001
	Clinical obesity	374	2.92 (2.55, 3.34)	<0.0001
	BMI-based obesity	682	3.71 (3.22, 4.27)	<0.0001
Glioma	No obesity	521	1.00	
	Preclinical obesity	134	0.82 (0.68, 1.00)	0.0884
	Clinical obesity	91	0.85 (0.68, 1.07)	0.2434
	BMI-based obesity	173	0.91 (0.74, 1.12)	0.5137
Head, neck, oral, pharynx, larynx	No obesity	850	1.00	
	Preclinical obesity	259	1.00 (0.87, 1.16)	0.9845
	Clinical obesity	167	0.91 (0.76, 1.08)	0.3533
	BMI-based obesity	318	0.79 (0.68, 0.92)	0.0060
Hodgkins lymphoma	No obesity	70	1.00	
	Preclinical obesity	33	1.35 (0.88, 2.06)	0.2434
	Clinical obesity	26	1.51 (0.94, 2.43)	0.1442
	BMI-based obesity	43	1.17 (0.74, 1.85)	0.6471
Kidney (renal cell)	No obesity	649	1.00	
	Preclinical obesity	306	1.47 (1.28, 1.69)	<0.0001
	Clinical obesity	250	1.76 (1.51, 2.05)	<0.0001
	BMI-based obesity	436	2.02 (1.71, 2.38)	<0.0001
Liver (hepatocellular carcinoma)	No obesity	143	1.00	
	Preclinical obesity	70	1.59 (1.18, 2.13)	0.0055
	Clinical obesity	102	2.96 (2.26, 3.88)	<0.0001
	BMI-based obesity	154	2.46 (1.78, 3.40)	<0.0001
Lung	No obesity	2257	1.00	
	Preclinical obesity	724	0.92 (0.85, 1.00)	0.1020
	Clinical obesity	792	1.17 (1.07, 1.27)	0.0013
	BMI-based obesity	993	0.84 (0.77, 0.92)	0.0007
Lymphoid leukemia	No obesity	483	1.00	
	Preclinical obesity	131	0.85 (0.69, 1.03)	0.1521
	Clinical obesity	107	0.96 (0.77, 1.19)	0.8388
	BMI-based obesity	188	1.00 (0.81, 1.23)	0.9995
Malignant melanoma	No obesity	1892	1.00	
	Preclinical obesity	581	1.00 (0.91, 1.10)	0.9845
	Clinical obesity	344	0.96 (0.85, 1.08)	0.5573
	BMI-based obesity	676	1.15 (1.04, 1.28)	0.0188
Multiple myeloma	No obesity	537	1.00	
	Preclinical obesity	186	1.12 (0.95, 1.33)	0.2521
	Clinical obesity	137	1.17 (0.96, 1.42)	0.1788
	BMI-based obesity	247	1.34 (1.11, 1.61)	0.0058
Myeloid leukemia	No obesity	259	1.00	
	Preclinical obesity	86	1.03 (0.80, 1.32)	0.9492
	Clinical obesity	73	1.20 (0.91, 1.57)	0.2708
	BMI-based obesity	112	1.24 (0.94, 1.63)	0.2217
Non-Hodgkins lymphoma	No obesity	863	1.00	
	Preclinical obesity	267	0.99 (0.86, 1.14)	0.9708
	Clinical obesity	184	0.98 (0.83, 1.15)	0.9085
	BMI-based obesity	328	1.03 (0.88, 1.20)	0.8588
Oesophagus (adenocarcinoma)	No obesity	369	1.00	
	Preclinical obesity	176	1.49 (1.24, 1.80)	<0.0001
	Clinical obesity	146	1.67 (1.36, 2.04)	<0.0001
	BMI-based obesity	252	1.99 (1.58, 2.50)	<0.0001
Oesophagus (squamous cell carcinoma)	No obesity	175	1.00	
	Preclinical obesity	31	0.55 (0.37, 0.81)	0.0063
	Clinical obesity	37	0.84 (0.58, 1.22)	0.4745
	BMI-based obesity	42	0.56 (0.38, 0.81)	0.0058
Ovary	No obesity	565	1.00	
	Preclinical obesity	222	1.15 (0.98, 1.35)	0.1442
	Clinical obesity	145	1.09 (0.90, 1.32)	0.4845
	BMI-based obesity	237	1.15 (0.96, 1.37)	0.2037
Pancreas	No obesity	702	1.00	
	Preclinical obesity	272	1.20 (1.04, 1.39)	0.0291
	Clinical obesity	238	1.45 (1.25, 1.70)	<0.0001
	BMI-based obesity	381	1.54 (1.31, 1.80)	<0.0001
Prostate (non-fatal)	No obesity	7299	1.00 (−)	
	Preclinical obesity	1840	0.91 (0.86, 0.95)	0.0007
	Clinical obesity	1193	0.88 (0.82, 0.93)	<0.0001
	BMI-based obesity	2310	0.88 (0.83, 0.94)	<0.0001
Prostate (fatal)	No obesity	496	1.00	
	Preclinical obesity	175	1.27 (1.06, 1.52)	0.0238
	Clinical obesity	117	1.21 (0.98, 1.50)	0.1394
	BMI-based obesity	207	1.14 (0.92, 1.41)	0.3306
Stomach (cardia)	No obesity	137	1.00	
	Preclinical obesity	61	1.47 (1.08, 2.00)	0.0344
	Clinical obesity	55	1.77 (1.27, 2.46)	0.0021
	BMI-based obesity	89	1.73 (1.20, 2.50)	0.0073
Stomach (non-cardia)	No obesity	112	1.00	
	Preclinical obesity	36	0.98 (0.67, 1.44)	0.9845
	Clinical obesity	25	0.82 (0.52, 1.29)	0.4845
	BMI-based obesity	42	0.68 (0.45, 1.04)	0.1320
Thyroid	No obesity	234	1.00	
	Preclinical obesity	96	1.34 (1.05, 1.70)	0.0412
	Clinical obesity	73	1.55 (1.17, 2.04)	0.0054
	BMI-based obesity	124	1.62 (1.24, 2.11)	0.0012
Obesity-related	No obesity	11,401	1.00	
	Preclinical obesity	4919	1.29 (1.25, 1.34)	<0.0001
	Clinical obesity	3611	1.34 (1.29, 1.39)	<0.0001
	BMI-based obesity	6095	1.47 (1.41, 1.53)	<0.0001
Overall	No obesity	29,567	1.00	
	Preclinical obesity	10,131	1.09 (1.06, 1.12)	<0.0001
	Clinical obesity	7362	1.13 (1.10, 1.16)	<0.0001
	BMI-based obesity	12,627	1.15 (1.12, 1.18)	<0.0001

BMI: Body mass index; FDR: False discovery rate.

The reference and overweight groups were included in the BMI-based obesity model but are not shown in the table.

Person-years of follow-up are not shown in the table: No obesity: 3,429,474; preclinical obesity: 1,013,746; clinical obesity: 649,183; BMI-based obesity: 1,240,349. Among women: No obesity: 1,772,106; preclinical obesity: 570,131; clinical obesity: 388,322; BMI-based obesity: 644,659.

Clinical obesity, as compared to no obesity, was associated with increased risk of 12 cancers (Table 4), including hepatocellular carcinoma and endometrial cancer (HRs 2.0–3.0), followed by cancers of the gastric cardia, kidney, biliary tract; esophageal adenocarcinoma; and thyroid cancer (HRs 1.5–2.0), as well as pancreatic, bladder, postmenopausal breast, colorectal, and lung cancers (HRs <1.5). Clinical obesity showed a stronger relation to obesity-related cancers combined (HR = 1.34; 95% CI = 1.29–1.39) than to all cancers combined (HR = 1.13; 95% CI = 1.10–1.16). It showed no associations with large B-cell lymphoma, fatal prostate cancer, and esophageal squamous cell carcinoma and was inversely related to non-fatal prostate cancer (Table 4).

Clinical obesity showed stronger associations than preclinical obesity with hepatocellular carcinoma, as well as endometrial, colorectal, pancreatic, and lung cancers. BMI-based obesity showed more pronounced associations than preclinical obesity with endometrial, postmenopausal breast, and kidney cancers. Additionally, BMI-based obesity was more strongly associated with postmenopausal breast cancer than clinical obesity. While clinical obesity was positively associated with lung cancer, BMI-based obesity showed an inverse relation (all p-difference <0.05) (Table 4, Table 5).

**Table 5:** Pairwise comparisons of preclinical obesity, clinical obesity, and BMI-based obesity in relation to cancer types.

Cancer type	Comparison group	FDR-adjusted p-value for difference
Biliary tract	Preclinical obesity vs. Clinical obesity	0.0523
	Preclinical obesity vs. BMI-based obesity	0.3450
	Clinical obesity vs. BMI-based obesity	0.0744
Bladder	Preclinical obesity vs. Clinical obesity	0.8047
	Preclinical obesity vs. BMI-based obesity	0.2900
	Clinical obesity vs. BMI-based obesity	0.3708
Breast (post-menopausal)	Preclinical obesity vs. Clinical obesity	0.7433
	Preclinical obesity vs. BMI-based obesity	0.0221
	Clinical obesity vs. BMI-based obesity	0.0216
Colorectum	Preclinical obesity vs. Clinical obesity	0.0386
	Preclinical obesity vs. BMI-based obesity	0.3297
	Clinical obesity vs. BMI-based obesity	0.8863
Endometrial	Preclinical obesity vs. Clinical obesity	0.0216
	Preclinical obesity vs. BMI-based obesity	0.0004
	Clinical obesity vs. BMI-based obesity	0.0085
Kidney (renal cell carcinoma)	Preclinical obesity vs. Clinical obesity	0.0596
	Preclinical obesity vs. BMI-based obesity	0.0377
	Clinical obesity vs. BMI-based obesity	0.2070
Liver (hepatocellular carcinoma)	Preclinical obesity vs. Clinical obesity	0.0009
	Preclinical obesity vs. BMI-based obesity	0.2000
	Clinical obesity vs. BMI-based obesity	0.7787
Lung	Preclinical obesity vs. Clinical obesity	<0.0001
	Preclinical obesity vs. BMI-based obesity	<0.0001
	Clinical obesity vs. BMI-based obesity	<0.0001
Oesophagus (adenocarcinoma)	Preclinical obesity vs. Clinical obesity	0.3745
	Preclinical obesity vs. BMI-based obesity	0.2929
	Clinical obesity vs. BMI-based obesity	0.5413
Oesophagus (squamous cell carcinoma)	Preclinical obesity vs. Clinical obesity	0.0587
	Preclinical obesity vs. BMI-based obesity	0.7504
	Clinical obesity vs. BMI-based obesity	0.1793
Pancreas	Preclinical obesity vs. Clinical obesity	0.0483
	Preclinical obesity vs. BMI-based obesity	0.0552
	Clinical obesity vs. BMI-based obesity	0.3584
Prostate (non-fatal)	Preclinical obesity vs. Clinical obesity	0.5033
	Preclinical obesity vs. BMI-based obesity	0.6904
	Clinical obesity vs. BMI-based obesity	0.9951
Prostate (fatal)	Preclinical obesity vs. Clinical obesity	0.7122
	Preclinical obesity vs. BMI-based obesity	0.1089
	Clinical obesity vs. BMI-based obesity	0.1830
Stomach (cardia)	Preclinical obesity vs. Clinical obesity	0.3325
	Preclinical obesity vs. BMI-based obesity	0.8904
	Clinical obesity vs. BMI-based obesity	0.5615
Thyroid	Preclinical obesity vs. Clinical obesity	0.3786
	Preclinical obesity vs. BMI-based obesity	0.5186
	Clinical obesity vs. BMI-based obesity	0.8357

BMI: Body mass index; FDR: False discovery rate.

When stratified by sex, clinical obesity was positively associated with colorectal cancer in men but not in women, while for multiple myeloma, it was positively associated in women but not in men (Supplemental Table S2). Additionally, clinical obesity had a stronger association with endometrial cancer in never-users than in ever-users of postmenopausal hormone therapy (all p-interaction <0.05) (Supplemental Table S3). Associations of preclinical and clinical obesity with cancer did not vary by age or smoking status (all p-interaction >0.05) (Supplemental Tables S4 and S5).

In sensitivity analyses that excluded the first two years of follow-up and underweight individuals, adjusted for ethnicity and family history of cancer, handled missing covariate data using multiple imputation (Supplemental Table S6), and restricted analyses to ICD-based organ dysfunction indicators (Supplemental Table S7), results were materially unchanged.

We estimated that, in the UK Biobank cohort, preclinical obesity accounted for 1.8% (1.3, 2.2) and clinical obesity for 1.7% (1.3, 2.1) of total cancer cases, while preclinical obesity accounted for 5.5% (4.7, 6.3) and clinical obesity for 4.3% (3.6, 4.9) of obesity-related cancers.

## Discussion

We examined the Lancet Commission's definitions of preclinical obesity (body fatness without organ dysfunction) and clinical obesity (with dysfunction),^2^ alongside the WHO's BMI-based definition,^14^ in relation to 28 malignancies. Nearly half showed positive associations with preclinical or clinical obesity, mainly affecting the digestive (esophageal adenocarcinoma, stomach, liver, biliary tract, pancreas, colorectum), reproductive (endometrium, postmenopausal breast, fatal prostate), urinary (bladder, kidney), respiratory (lung), and endocrine (thyroid) systems.

For most cancers, our findings align with those of the IARC^5^ and the WCRF,^6^ which primarily rely on BMI to measure body fatness. However, some discrepancies emerged. We found no relations of preclinical and clinical obesity to ovarian cancer, consistent with our BMI-based obesity results, despite IARC or WCRF classifying the evidence for this malignancy as sufficient or probable. Conversely, we found that clinical obesity was positively associated with lung cancer and inversely related to esophageal squamous cell carcinoma, cancers for which IARC and WCRF consider the evidence limited or inadequate. In a sub-analysis, we observed a positive association between clinical obesity and multiple myeloma in women, consistent with IARC's classification of the evidence for this cancer as sufficient.

Obesity-driven carcinogenesis involves dysregulated lipid metabolism, insulin resistance, inflammation, and adipokine signaling, mechanisms that collectively promote tumor proliferation, migration, angiogenesis, and reduced apoptosis.^20^ Our findings suggest that cancer risk arises from both excess adiposity and its metabolic and organ-related effects, highlighting the value of distinguishing obesity subtypes in risk assessment, although the specific advantages of such distinctions remain to be determined. At the same time, it is important to emphasize that clinical obesity and cancer are distinct disease entities, each with their own diagnostic criteria, clinical course, and underlying pathophysiology.

Elaborating on these results, our findings show that preclinical obesity was related to increased cancer risk compared to no obesity, pointing to a link beyond abnormalities detected by standard metabolic or organ function tests, such as hyperglycemia, dyslipidemia, fatty liver, or reproductive dysfunction (e.g., anovulation, male hypogonadism). These findings indicate that subclinical effects of excess adipose tissue, such as elevated leptin, TNF-α, IL-6, estrogen, and reduced testosterone, may drive tumorigenesis without overt clinical dysfunction.

By comparison, clinical obesity showed stronger associations than preclinical obesity, particularly with hepatocellular carcinoma and endometrial, colorectal, pancreatic, and lung cancers. These stronger associations may reflect additive effects of both excess adiposity and organ dysfunction. Organ-specific impairments, such as hepatic steatosis, insulin resistance, or chronic inflammation, could exacerbate biological processes like cellular proliferation, immune evasion, or impaired DNA repair, thereby amplifying cancer risk beyond that associated with adiposity alone. For example, hepatic dysfunction in fatty liver disease may promote hepatocellular carcinoma through chronic inflammation and compensatory hepatocyte proliferation; insulin resistance may drive endometrial cancer via hormonal imbalances that favor estrogen signaling; metabolic dysfunction may promote colorectal cancer by enhancing insulin/IGF-1 activity and epithelial proliferation; systemic inflammation associated with metabolic disease may foster a pro-tumorigenic environment in the pancreas; and respiratory dysfunction may exacerbate hypoxia and oxidative stress in lung tissue.

Although both preclinical and clinical obesity were related to elevated cancer risk, the role of obesity in cancer is indirect and probabilistic.^2^ Excess adiposity increases cancer risk but does not cause malignancy per se. Carcinogenesis typically requires additional biological triggers, such as genomic instability and immune evasion.^4^ These hallmark mechanisms differ fundamentally from the metabolic and functional impairments that define clinical obesity, underscoring that these are distinct pathological processes. Recognizing this distinction emphasizes that obesity serves as a modifiable risk factor, not an early stage of malignancy.

In terms of the proportion of cancers attributable to excess adiposity, the sizeable impact of preclinical obesity (5.5%) compared to clinical obesity (4.3%) on obesity-related cancer suggests that early-stage excess weight contributes substantially to the population cancer burden, underscoring the importance of early weight management as a public health priority.

It is worth noting that BMI-based obesity showed stronger associations than preclinical-clinical classifications of obesity for several cancer types. This is likely due to reference group differences and similar body mass composition in preclinical and clinical obesity. First, the BMI-based reference group included only normal-weight individuals, whereas nearly 50% of the preclinical and clinical obesity reference group was overweight, likely dampening risk estimates for cancers where overweight increases risk (e.g., breast, colorectal, endometrial, and kidney cancers) and elevating them where it decreases risk (e.g., lung cancer). Second, similar BMI distributions in preclinical and clinical obesity led to comparable risk magnitudes (e.g., breast cancer) or non-linear patterns (e.g., colorectal cancer).

Given that overweight individuals were allocated to the reference, preclinical obesity, and clinical obesity groups, minimizing body mass differences between these groups, cancer risk increased notably with progression from no obesity to preclinical and clinical stages, likely reflecting the sustained impact of excess adiposity and organ dysfunction.

A notable finding was the contrasting associations with lung cancer. BMI-based obesity displayed an inverse association, likely due to residual confounding by smoking, as smoking is associated with lower adiposity and higher lung cancer risk.^21^ In contrast, clinical obesity showed a positive relation, likely because it captures risk related to both excess fat and smoking-induced respiratory dysfunction, making it less prone to smoking confounding. Mendelian randomization evidence supports a causal positive association between BMI and lung cancer,^22^ reinforcing the biological link between adiposity and bronchial tumorigenesis.

A similar but weaker pattern was observed for esophageal squamous cell carcinoma. Both preclinical obesity and BMI-based obesity showed strong inverse associations, likely due to residual confounding by smoking, as seen with lung cancer. In contrast, clinical obesity showed a weak, non-significant inverse relation. Unlike the other measures, clinical obesity accounts for organ dysfunction markers linked to alcohol use and smoking, such as elevated liver function tests and respiratory issues, potentially shifting the risk estimate upward toward the null value. Residual confounding by alcohol intake may also have contributed to this shift, as clinical obesity was related to the lowest current alcohol consumption. While no Mendelian randomization studies have examined the relation of obesity to esophageal squamous cell carcinoma, a meta-analysis of 25 prospective studies suggests an inverse association.^23^

Preclinical obesity was specifically associated with increased fatal prostate cancer risk, whereas neither clinical obesity nor BMI-based obesity were significant. This may reflect the role of central adiposity, better captured by waist circumference, in elevating insulin, inflammatory cytokines, and growth factors that promote aggressive tumor behaviour.^24^ The absence of an association with clinical obesity suggests that metabolic conditions like diabetes may offset obesity's adverse effects on aggressive disease. Conversely, all obesity classifications were associated with reduced non-fatal prostate cancer risk, suggesting a dominant role of lower testosterone in non-aggressive cases. Mendelian randomization analyses suggest that adiposity lowers bioavailable testosterone,^25^ supporting our observed inverse association with non-fatal cases. These interpretations are based on plausible biological mechanisms proposed in the literature; specific mechanistic pathways were not directly assessed in our study.

Our stratified sub-analyses revealed sex-specific variations in obesity-cancer associations. The stronger association observed between clinical obesity and colorectal cancer in men compared to women aligns with prior research and may reflect the notion that adulthood obesity captures risk more accurately in men, for whom adult weight gain is a key risk factor for colorectal cancer. In contrast, early-life obesity appears to be more influential in women.^26^ The more pronounced association between clinical obesity and multiple myeloma in women compared to men is consistent with previous research showing a stronger link between adiposity and multiple myeloma mortality in women.^27^ One potential underlying mechanism involves low adiponectin levels,^28^ which relate inversely to body fat in men but, to a stronger degree, with metabolic and hormonal factors in women,^29^ potentially intensifying the adverse impact of obesity on multiple myeloma risk in women. However, sex-specific biologic mechanisms were not directly investigated in our study and should be interpreted as plausible rather than demonstrated pathways.

In analyses stratified by hormone use, the stronger association between obesity and endometrial cancer in never users of postmenopausal hormones compared to ever users is consistent with prior research^30^ and suggests that unopposed estrogen in postmenopausal hormone therapy may attenuate the relative influence of adipose-derived estrogen on endometrial carcinogenesis.

Our findings suggest that BMI-based and preclinical-clinical classifications of obesity serve complementary rather than competing purposes. While BMI remains a simple and practical tool for population-level monitoring, the Lancet Commission framework offers a more biologically grounded classification that incorporates organ dysfunction and mobility limitations, potentially enhancing individualized risk assessment. Future research should explore under what conditions and in which populations the preclinical-clinical framework yields superior predictive power for cancer risk or progression. Hybrid models integrating BMI with clinical markers of dysfunction may help optimize stratification for targeted intervention strategies.

The preclinical-clinical obesity framework may also support earlier and more tailored cancer prevention strategies. Individuals with preclinical obesity represent a window of opportunity for early intervention, where lifestyle modification and weight management could reduce cancer risk before physiological systems become impaired. Public health policies could integrate this stratification to prioritize preventive outreach and behavioral programs for at-risk individuals, even in the absence of clinical disease. Clinically, the framework can be operationalized using existing tools such as BMI, waist circumference, and routine health records to screen for signs of organ dysfunction. Embedding this approach into primary care workflows could help differentiate those needing intensive metabolic monitoring or referral from those who might benefit from community-based weight management support.

Key strengths of our study include its large sample size, providing robust statistical power, and its prospective design, which minimized reverse causation, recall bias, and selection bias. In addition, detailed subgroup and sensitivity analyses identified potential effect modification and confirmed the robustness of our findings. Moreover, the stringent correction for multiple comparisons led to measured conclusions, enhancing credibility and establishing a solid foundation for future research, where replication can further strengthen confidence in the observed associations.

Limitations arise from both the conceptual framework and the methodological challenges of applying preclinical and clinical obesity classifications in epidemiologic research. Most broadly, the classifications do not distinguish fat from lean mass or visceral from subcutaneous fat, differences that are relevant to cancer risk. Additionally, defining clinical obesity requires diagnosed organ dysfunction, which can be difficult to ascertain in epidemiologic settings due to variability in and limited availability of diagnostic measures.

In this study, most classifications were based on ICD codes, although self-reported data were used to capture musculoskeletal dysfunction. However, the diagnostic accuracy of electronic health records may vary depending on coding practices and access to diagnostic testing, potentially affecting the validity of ICD-based classifications. Self-reported mobility limitations may also have been incompletely or inaccurately captured. Furthermore, longitudinal data on adiposity and organ dysfunction were lacking, preventing observation of transitions from no excess body fat to preclinical or clinical obesity, or from preclinical to clinical obesity. These limitations may have introduced a degree of non-differential exposure misclassification, which typically biases results toward the null and could have led to underestimation of cancer risk associated with clinical obesity.

An additional concern relates to the potential for collider bias stemming from the definition of clinical obesity, which, unlike preclinical obesity, was based on the presence of organ dysfunction. Such bias could arise if organ dysfunction was influenced by both abnormal fat mass and preclinical malignancy, or if analyses were restricted to individuals included in electronic health records, and health care utilization was influenced by factors related to both obesity and cancer. In these scenarios, collider bias could have resulted in spurious or exaggerated associations. However, this type of bias is unlikely to have meaningfully distorted our findings, as clinical obesity was more strongly associated with most cancer types than preclinical obesity, which was not subject to this bias. Such a consistent pattern across diverse cancer sites would be unlikely to arise solely from collider bias, which would require highly similar and specific underlying structures across all relevant variables.

A further potential shortcoming is that, although the UK Biobank provides a large and richly phenotyped sample, it is not fully representative of the UK population. Participants tend to be healthier, more affluent, and less ethnically diverse, which may have led to conservative estimates of associations and may limit generalizability.

Future research should prioritize longitudinal designs with repeated assessments, including clinical evaluations, biomarkers, imaging, and functional measures, to examine the validity and reproducibility of the clinical obesity phenotype and to elucidate causal mechanisms linking obesity subtypes to site-specific cancer development and prognosis. To strengthen generalizability, prospective studies in independent cohorts are warranted to test the broader applicability of the Lancet classification in predicting cancer risk and progression. In addition, causal mediation analysis should be used to quantify the direct and indirect effects of obesity on cancer risk.

In conclusion, preclinical obesity was related to increased cancer risk even in the absence of organ dysfunction, suggesting that obesity-related carcinogenesis extends beyond clinically detectable abnormalities. Clinical obesity conferred greater cancer risk, reinforcing the role of organ dysfunction in exacerbating carcinogenesis. Both preclinical and clinical obesity showed weaker associations than BMI-based obesity for several cancers, likely because both the reference and comparison groups for preclinical and clinical obesity contained a sizeable proportion of overweight individuals, minimizing body mass differences between these groups and attenuating risk estimates. Our findings highlight the complexity of obesity-related cancer risk, underscoring the need to refine obesity classification to better capture its biological, clinical, and population-level heterogeneity. Further research should determine whether these classifications improve cancer risk assessment in specific contexts.

## Contributors

MJS and MFL had full access to all the data in the study and take responsibility for the integrity of the data and the accuracy of the data analysis. Study design: all authors; Acquisition, analysis, or interpretation of the data: all authors; Manuscript writing: all authors; Critical revision of the manuscript for important intellectual content: all authors.

## Data sharing statement

UK Biobank is an open access resource. Bona fide researchers can apply to use the UK Biobank dataset by registering and applying at http://ukbiobank.ac.uk/register-apply/.

## Declaration of interests

All authors disclose no conflict of interest for this work.
