# Supplementary material for: Excess adiposity and cancer: evaluating a preclinical-clinical obesity framework for risk stratification
Source: eClinicalMedicine. 2025 May 12;83:103247. doi: 10.1016/j.eclinm.2025.103247 (PMC12140032; doi:10.1016/j.eclinm.2025.103247)
Supplement: Supplementary Figure and Tables [file mmc1.docx]

**Excess Adiposity and Cancer: Evaluating a Preclinical-Clinical Obesity Framework for Risk Stratification**

Supplemental material

Content

Supplemental Figure 1: Flowchart of participant exclusions

# Supplemental Table 1: Numbers of cancer cases

# Supplemental Table 2: Hazard ratios and 95% confidence intervals for preclinical obesity and clinical obesity in relation to cancer types according to sex

# Supplemental Table 3: Hazard ratios and 95% confidence intervals for preclinical obesity and clinical obesity in relation to female-specific cancer types according to postmenopausal hormone use

# Supplemental Table 4: Hazard ratios and 95% confidence intervals for preclinical obesity and clinical obesity in relation to cancer types according to age

# Supplemental Table 5: Hazard ratios and 95% confidence intervals for preclinical obesity and clinical obesity in relation to smoking-related cancer types according to smoking status

# Supplemental Table 6: Hazard ratios and 95% confidence intervals for preclinical obesity and clinical obesity in relation to cancer types after excluding the first two years of follow-up and underweight individuals, adjusting for ethnicity and family history of cancer, and handling missing covariate data using multiple imputation

# Supplemental Table 7: Hazard ratios and 95% confidence intervals for preclinical obesity and clinical obesity in relation to cancer types after restricting analyses to ICD-based organ dysfunction indicators

# Supplemental Figure 1: Flowchart of participant exclusions

#
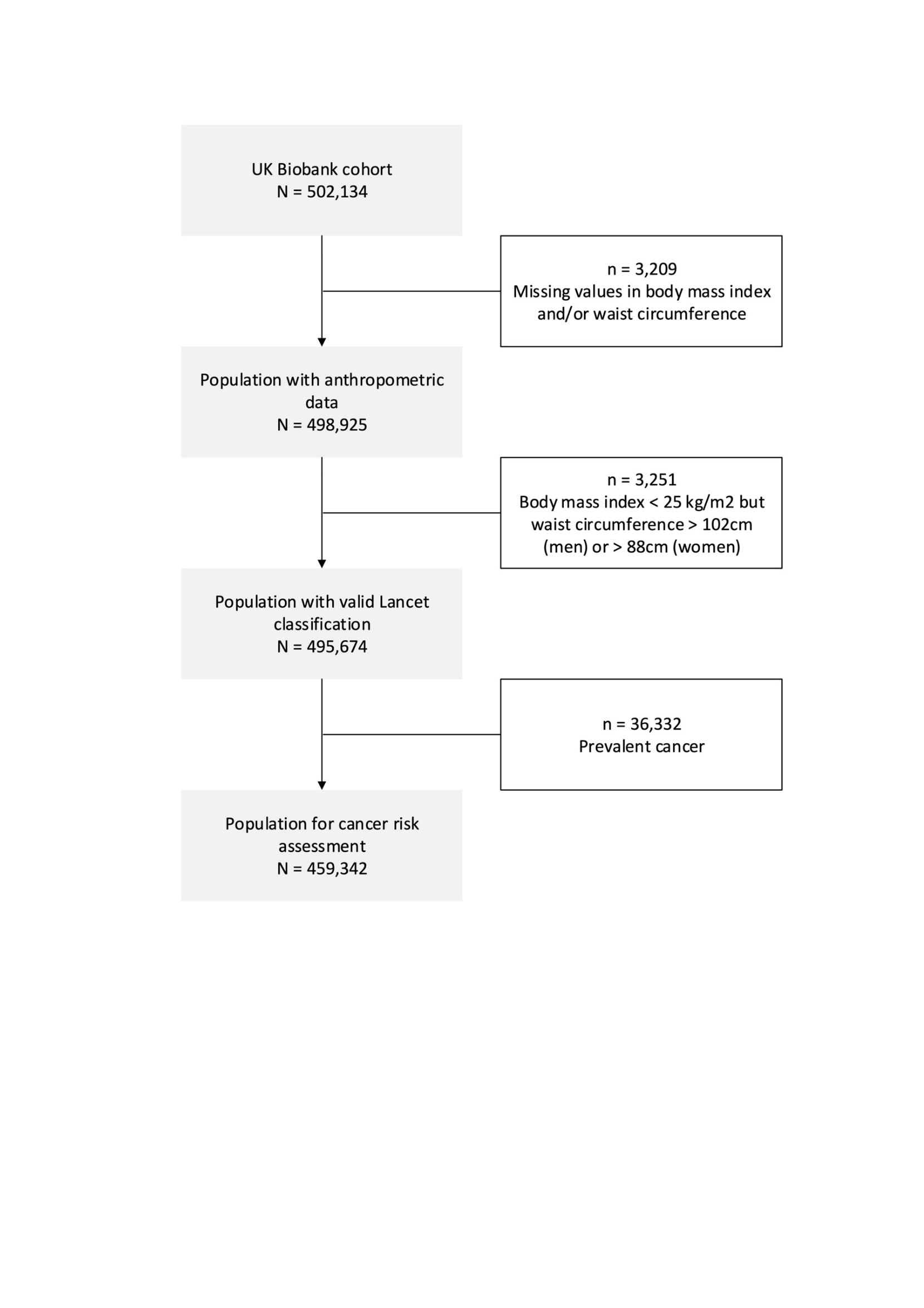


# Supplemental Table 1: Numbers of cancer cases

| **Cancer** | **No obesity** | **Preclinical obesity** | **Clinical obesity** |
| --- | --- | --- | --- |
|  | **N = 29,786** | **N = 10,210** | **N = 7,402** |
| Biliary tract | 282 (0.9%) | 123 (1.2%) | 123 (1.7%) |
| Bladder | 655 (2.2%) | 248 (2.4%) | 188 (2.5%) |
| Breast (post-menopausal) | 3,723 (12.5%) | 1,583 (15.5%) | 1,120 (15.1%) |
| Breast (pre-menopausal) | 1,710 (5.7%) | 474 (4.6%) | 272 (3.7%) |
| Cervix | 77 (0.3%) | 24 (0.2%) | 16 (0.2%) |
| Colorectum | 3,489 (11.7%) | 1,361 (13.3%) | 860 (11.6%) |
| Diffuse large B-cell lymphoma | 472 (1.6%) | 177 (1.7%) | 122 (1.6%) |
| Endometrial | 627 (2.1%) | 493 (4.8%) | 375 (5.1%) |
| Glioma | 524 (1.8%) | 135 (1.3%) | 92 (1.2%) |
| Head, neck, oral, pharynx, larynx | 858 (2.9%) | 263 (2.6%) | 169 (2.3%) |
| Hodgkins lymphoma | 71 (0.2%) | 33 (0.3%) | 26 (0.4%) |
| Kidney (renal cell carcinoma) | 651 (2.2%) | 309 (3.0%) | 252 (3.4%) |
| Liver (hepatocellular carcinoma) | 145 (0.5%) | 70 (0.7%) | 102 (1.4%) |
| Lung | 2,281 (7.7%) | 730 (7.1%) | 796 (10.8%) |
| Lymphoid leukemia | 485 (1.6%) | 131 (1.3%) | 107 (1.4%) |
| Malignant melanoma | 1,909 (6.4%) | 582 (5.7%) | 345 (4.7%) |
| Multiple myeloma | 544 (1.8%) | 185 (1.8%) | 138 (1.9%) |
| Myeloid leukemia | 259 (0.9%) | 86 (0.8%) | 73 (1.0%) |
| Non-Hodgkins lymphoma | 867 (2.9%) | 268 (2.6%) | 186 (2.5%) |
| Oesophagus (adenocarcinoma) | 370 (1.2%) | 176 (1.7%) | 147 (2.0%) |
| Oesophagus (squamous cell carcinoma) | 178 (0.6%) | 31 (0.3%) | 37 (0.5%) |
| Ovary | 571 (1.9%) | 224 (2.2%) | 146 (2.0%) |
| Pancreas | 713 (2.4%) | 276 (2.7%) | 240 (3.2%) |
| Prostate (non-fatal) | 7,346 (24.7%) | 1,857 (18.2%) | 1,198 (16.2%) |
| Prostate (fatal) | 496 (1.7%) | 175 (1.7%) | 117 (1.6%) |
| Stomach (cardia) | 137 (0.5%) | 63 (0.6%) | 56 (0.8%) |
| Stomach (non-cardia) | 112 (0.4%) | 37 (0.4%) | 25 (0.3%) |
| Thyroid | 234 (0.8%) | 96 (0.9%) | 74 (1.0%) |

#

# Supplemental Table 2: Hazard ratios and 95% confidence intervals for preclinical obesity and clinical obesity in relation to cancer types according to sex

| **Cancer type** | **Group** | **Women** | |  | **Men** | | **FDR-adjusted**  **P-value for interaction** |
| --- | --- | --- | --- | --- | --- | --- | --- |
|  |  | **Cases** | **HR (95% CI)** |  | **Cases** | **HR (95% CI)** |  |
| Biliary tract | No obesity | 131 | 1.00 |  | 146 | 1.00 |  |
|  | Preclinical obesity | 64 | 1.29 (0.95, 1.75) |  | 57 | 1.35 (0.98, 1.85) | 0.9631 |
|  | Clinical obesity | 67 | 1.76 (1.29, 2.40) |  | 53 | 1.65 (1.19, 2.30) | 0.8869 |
| Bladder | No obesity | 133 | 1.00 |  | 518 | 1.00 |  |
|  | Preclinical obesity | 56 | 1.10 (0.80, 1.52) |  | 189 | 1.19 (1.00, 1.41) | 0.8869 |
|  | Clinical obesity | 49 | 1.23 (0.88, 1.74) |  | 138 | 1.21 (0.99, 1.47) | 0.8869 |
| Colorectum | No obesity | 1,456 | 1.00 |  | 2,001 | 1.00 |  |
|  | Preclinical obesity | 602 | 1.18 (1.07, 1.30) |  | 746 | 1.27 (1.17, 1.39) | 0.5369 |
|  | Clinical obesity | 364 | 0.99 (0.88, 1.11) |  | 492 | 1.23 (1.11, 1.36) | 0.0270 |
| Diffuse large B-cell lymphoma | No obesity | 189 | 1.00 |  | 281 | 1.00 |  |
|  | Preclinical obesity | 96 | 1.42 (1.10, 1.82) |  | 81 | 1.03 (0.80, 1.33) | 0.2831 |
|  | Clinical obesity | 64 | 1.28 (0.95, 1.72) |  | 58 | 1.09 (0.81, 1.46) | 0.7476 |
| Glioma | No obesity | 202 | 1.00 |  | 319 | 1.00 |  |
|  | Preclinical obesity | 67 | 0.96 (0.72, 1.27) |  | 67 | 0.71 (0.54, 0.94) | 0.4845 |
|  | Clinical obesity | 34 | 0.67 (0.46, 0.98) |  | 57 | 1.00 (0.74, 1.34) | 0.5432 |
| Head, neck, oral, pharynx, larynx | No obesity | 239 | 1.00 |  | 611 | 1.00 |  |
|  | Preclinical obesity | 93 | 1.13 (0.89, 1.45) |  | 166 | 0.96 (0.80, 1.14) | 0.7088 |
|  | Clinical obesity | 54 | 0.90 (0.67, 1.23) |  | 113 | 0.93 (0.75, 1.14) | 0.8869 |
| Hodgkins lymphoma | No obesity | 35 | 1.00 |  | 35 | 1.00 |  |
|  | Preclinical obesity | 16 | 1.25 (0.68, 2.29) |  | 17 | 1.52 (0.84, 2.76) | 0.8869 |
|  | Clinical obesity | 9 | 0.93 (0.43, 1.99) |  | 17 | 2.30 (1.24, 4.27) | 0.2760 |
| Kidney (renal cell carcinoma) | No obesity | 210 | 1.00 |  | 439 | 1.00 |  |
|  | Preclinical obesity | 111 | 1.47 (1.16, 1.86) |  | 195 | 1.46 (1.23, 1.74) | 0.9561 |
|  | Clinical obesity | 101 | 1.83 (1.43, 2.35) |  | 149 | 1.71 (1.41, 2.08) | 0.8869 |
| Liver (hepatocellular carcinoma) | No obesity | 30 | 1.00 |  | 113 | 1.00 |  |
|  | Preclinical obesity | 12 | 0.98 (0.49, 1.94) |  | 58 | 1.77 (1.28, 2.45) | 0.5520 |
|  | Clinical obesity | 13 | 1.24 (0.62, 2.47) |  | 89 | 3.52 (2.62, 4.73) | 0.1440 |
| Lung | No obesity | 1,002 | 1.00 |  | 1,255 | 1.00 |  |
|  | Preclinical obesity | 369 | 0.95 (0.84, 1.08) |  | 355 | 0.89 (0.79, 1.00) | 0.8869 |
|  | Clinical obesity | 425 | 1.25 (1.11, 1.40) |  | 367 | 1.09 (0.96, 1.22) | 0.6433 |
| Lymphoid leukemia | No obesity | 175 | 1.00 |  | 308 | 1.00 |  |
|  | Preclinical obesity | 54 | 0.86 (0.63, 1.17) |  | 77 | 0.84 (0.65, 1.08) | 0.9561 |
|  | Clinical obesity | 43 | 0.91 (0.64, 1.28) |  | 64 | 1.00 (0.75, 1.32) | 0.8869 |
| Malignant melanoma | No obesity | 915 | 1.00 |  | 977 | 1.00 |  |
|  | Preclinical obesity | 276 | 0.92 (0.80, 1.06) |  | 305 | 1.08 (0.95, 1.24) | 0.2831 |
|  | Clinical obesity | 168 | 0.86 (0.72, 1.02) |  | 176 | 1.06 (0.90, 1.25) | 0.2760 |
| Multiple myeloma | No obesity | 209 | 1.00 |  | 328 | 1.00 |  |
|  | Preclinical obesity | 73 | 1.01 (0.77, 1.33) |  | 113 | 1.22 (0.98, 1.52) | 0.5777 |
|  | Clinical obesity | 85 | 1.56 (1.20, 2.04) |  | 52 | 0.84 (0.62, 1.14) | 0.0330 |
| Myeloid leukemia | No obesity | 99 | 1.00 |  | 160 | 1.00 |  |
|  | Preclinical obesity | 47 | 1.30 (0.91, 1.86) |  | 39 | 0.82 (0.58, 1.18) | 0.2831 |
|  | Clinical obesity | 32 | 1.21 (0.80, 1.83) |  | 41 | 1.21 (0.84, 1.73) | 0.9561 |
| Non-Hodgkins lymphoma | No obesity | 386 | 1.00 |  | 477 | 1.00 |  |
|  | Preclinical obesity | 136 | 1.01 (0.83, 1.23) |  | 131 | 0.97 (0.79, 1.18) | 0.8869 |
|  | Clinical obesity | 80 | 0.81 (0.63, 1.03) |  | 104 | 1.16 (0.93, 1.44) | 0.2831 |
| Oesophagus (adenocarcinoma) | No obesity | 56 | 1.00 |  | 313 | 1.00 |  |
|  | Preclinical obesity | 30 | 1.41 (0.90, 2.22) |  | 146 | 1.52 (1.24, 1.85) | 0.8869 |
|  | Clinical obesity | 26 | 1.61 (0.99, 2.62) |  | 120 | 1.68 (1.35, 2.09) | 0.8869 |
| Oesophagus (squamous cell carcinoma) | No obesity | 97 | 1.00 |  | 78 | 1.00 |  |
|  | Preclinical obesity | 18 | 0.53 (0.32, 0.88) |  | 13 | 0.60 (0.33, 1.09) | 0.8869 |
|  | Clinical obesity | 20 | 0.74 (0.45, 1.22) |  | 17 | 1.01 (0.58, 1.74) | 0.5638 |
| Pancreas | No obesity | 303 | 1.00 |  | 399 | 1.00 |  |
|  | Preclinical obesity | 138 | 1.27 (1.03, 1.56) |  | 134 | 1.14 (0.93, 1.39) | 0.8869 |
|  | Clinical obesity | 117 | 1.40 (1.12, 1.75) |  | 121 | 1.50 (1.22, 1.86) | 0.8869 |
| Stomach (cardia) | No obesity | 22 | 1.00 |  | 115 | 1.00 |  |
|  | Preclinical obesity | 9 | 1.10 (0.50, 2.43) |  | 52 | 1.55 (1.10, 2.17) | 0.8207 |
|  | Clinical obesity | 16 | 2.51 (1.27, 4.96) |  | 39 | 1.58 (1.08, 2.31) | 0.5848 |
| Stomach (non-cardia) | No obesity | 43 | 1.00 |  | 69 | 1.00 |  |
|  | Preclinical obesity | 15 | 0.96 (0.53, 1.75) |  | 21 | 1.01 (0.61, 1.67) | 0.8869 |
|  | Clinical obesity | 8 | 0.61 (0.28, 1.32) |  | 17 | 0.99 (0.57, 1.72) | 0.5638 |
| Thyroid | No obesity | 168 | 1.00 |  | 66 | 1.00 |  |
|  | Preclinical obesity | 70 | 1.32 (0.99, 1.75) |  | 26 | 1.37 (0.86, 2.19) | 0.8869 |
|  | Clinical obesity | 58 | 1.65 (1.20, 2.26) |  | 15 | 1.25 (0.69, 2.25) | 0.8869 |
| Obesity-related | No obesity | 7,481 | 1.00 |  | 3,920 | 1.00 |  |
|  | Preclinical obesity | 3,392 | 1.28 (1.23, 1.33) |  | 1,527 | 1.32 (1.24, 1.40) | 0.5728 |
|  | Clinical obesity | 2,481 | 1.31 (1.24, 1.37) |  | 1,130 | 1.42 (1.32, 1.52) | 0.0270 |
| Overall | No obesity | 12,764 | 1.00 |  | 16,803 | 1.00 |  |
|  | Preclinical obesity | 5,128 | 1.16 (1.13, 1.20) |  | 5,003 | 1.03 (1.00, 1.06) | <0.0001 |
|  | Clinical obesity | 3,753 | 1.19 (1.15, 1.24) |  | 3,609 | 1.08 (1.04, 1.12) | 0.4831 |
| FDR: false discovery rate.  FDR was applied for each classification separately. | | | | | | | |

# Supplemental Table 3: Hazard ratios and 95% confidence intervals for preclinical obesity and clinical obesity in relation to female-specific cancer types according to postmenopausal hormone use

| **Cancer type** | **Group** | **Never used**  **postmenopausal hormones** | |  | **Ever used**  **postmenopausal hormones** | | **FDR-**  **adjusted**  **P-value for interaction** |
| --- | --- | --- | --- | --- | --- | --- | --- |
|  |  | **Cases** | **HR (95% CI)** |  | **Cases** | **HR (95% CI)** |  |
| Breast (pre-menopausal) | No obesity | 1,578 | 1.00 |  | 106 | 1.00 |  |
|  | Preclinical obesity | 422 | 1.01 (0.90, 1.12) |  | 46 | 1.45 (1.02, 2.07) | 0.2015 |
|  | Clinical obesity | 228 | 1.02 (0.88, 1.17) |  | 41 | 1.42 (0.97, 2.08) | 0.2015 |
| Breast (post-menopausal) | No obesity | 1,721 | 1.00 |  | 1,974 | 1.00 |  |
|  | Preclinical obesity | 761 | 1.17 (1.07, 1.27) |  | 801 | 1.19 (1.09, 1.29) | 0.8995 |
|  | Clinical obesity | 466 | 1.21 (1.09, 1.34) |  | 644 | 1.13 (1.03, 1.24) | 0.4637 |
| Endometrial | No obesity | 371 | 1.00 |  | 249 | 1.00 |  |
|  | Preclinical obesity | 332 | 2.63 (2.26, 3.06) |  | 147 | 1.83 (1.49, 2.25) | 0.0150 |
|  | Clinical obesity | 233 | 3.12 (2.62, 3.70) |  | 141 | 2.07 (1.67, 2.57) | 0.0140 |
| Ovary | No obesity | 323 | 1.00 |  | 239 | 1.00 |  |
|  | Preclinical obesity | 134 | 1.21 (0.98, 1.48) |  | 84 | 1.02 (0.79, 1.31) | 0.4637 |
|  | Clinical obesity | 67 | 1.00 (0.76, 1.31) |  | 78 | 1.11 (0.85, 1.44) | 0.7515 |
| Female cancers combined | No obesity | 3,993 | 1.00 |  | 2,568 | 1.00 |  |
|  | Preclinical obesity | 1,649 | 1.25 (1.18, 1.33) |  | 1,078 | 1.24 (1.15, 1.34) | 0.8995 |
|  | Clinical obesity | 994 | 1.31 (1.22, 1.41) |  | 904 | 1.23 (1.14, 1.33) | 0.4637 |
| FDR: false discovery rate.  FDR was applied for each classification separately. | | | | | | | |

# Supplemental Table 4: Hazard ratios and 95% confidence intervals for preclinical obesity and clinical obesity in relation to cancer types according to age

| **Cancer type** | **Group** | **<60 years** | |  | **≥60 years** | | **FDR-adjusted**  **P-value for interaction** |
| --- | --- | --- | --- | --- | --- | --- | --- |
|  |  | **Cases** | **HR (95% CI)** |  | **Cases** | **HR (95% CI)** |  |
| Biliary tract | No obesity | 83 | 1.00 |  | 194 | 1.00 |  |
|  | Preclinical obesity | 46 | 1.87 (1.29, 2.71) |  | 75 | 1.09 (0.83, 1.43) | 0.3360 |
|  | Clinical obesity | 30 | 2.10 (1.36, 3.26) |  | 90 | 1.55 (1.19, 2.02) | 0.8745 |
| Bladder | No obesity | 187 | 1.00 |  | 464 | 1.00 |  |
|  | Preclinical obesity | 64 | 1.12 (0.83, 1.49) |  | 181 | 1.21 (1.02, 1.45) | 0.9179 |
|  | Clinical obesity | 26 | 0.80 (0.52, 1.22) |  | 161 | 1.37 (1.13, 1.65) | 0.3360 |
| Breast (post-menopausal) | No obesity | 1,447 | 1.00 |  | 2,262 | 1.00 |  |
|  | Preclinical obesity | 559 | 1.18 (1.07, 1.31) |  | 1,014 | 1.20 (1.11, 1.29) | 0.9860 |
|  | Clinical obesity | 358 | 1.19 (1.06, 1.35) |  | 757 | 1.18 (1.08, 1.28) | 0.9179 |
| Cervix | No obesity | 51 | 1.00 |  | 26 | 1.00 |  |
|  | Preclinical obesity | 14 | 0.92 (0.50, 1.68) |  | 9 | 0.90 (0.41, 2.01) | 0.5625 |
|  | Clinical obesity | 9 | 1.00 (0.48, 2.09) |  | 7 | 1.00 (0.41, 2.45) | 0.3360 |
| Colorectum | No obesity | 1,360 | 1.00 |  | 2,097 | 1.00 |  |
|  | Preclinical obesity | 484 | 1.17 (1.06, 1.31) |  | 864 | 1.26 (1.17, 1.37) | 0.9867 |
|  | Clinical obesity | 232 | 1.03 (0.89, 1.19) |  | 624 | 1.17 (1.06, 1.28) | 0.9951 |
| Diffuse large B-cell lymphoma | No obesity | 168 | 1.00 |  | 302 | 1.00 |  |
|  | Preclinical obesity | 51 | 1.05 (0.76, 1.44) |  | 126 | 1.27 (1.03, 1.58) | 0.9867 |
|  | Clinical obesity | 38 | 1.36 (0.94, 1.97) |  | 84 | 1.10 (0.86, 1.42) | 0.7617 |
| Endometrial | No obesity | 318 | 1.00 |  | 304 | 1.00 |  |
|  | Preclinical obesity | 228 | 2.32 (1.95, 2.77) |  | 260 | 2.36 (1.99, 2.80) | 0.6572 |
|  | Clinical obesity | 146 | 2.65 (2.16, 3.27) |  | 228 | 3.12 (2.60, 3.73) | 0.5625 |
| Glioma | No obesity | 257 | 1.00 |  | 264 | 1.00 |  |
|  | Preclinical obesity | 63 | 0.83 (0.63, 1.10) |  | 71 | 0.83 (0.63, 1.08) | 0.6501 |
|  | Clinical obesity | 35 | 0.87 (0.61, 1.26) |  | 56 | 0.85 (0.63, 1.14) | 0.6501 |
| Head, neck, oral, pharynx, larynx | No obesity | 464 | 1.00 |  | 386 | 1.00 |  |
|  | Preclinical obesity | 132 | 0.99 (0.81, 1.20) |  | 127 | 1.02 (0.83, 1.25) | 0.9673 |
|  | Clinical obesity | 72 | 0.91 (0.70, 1.17) |  | 95 | 0.90 (0.71, 1.14) | 0.5480 |
| Hodgkins lymphoma | No obesity | 36 | 1.00 |  | 34 | 1.00 |  |
|  | Preclinical obesity | 15 | 1.29 (0.70, 2.40) |  | 18 | 1.37 (0.76, 2.46) | 0.9179 |
|  | Clinical obesity | 12 | 1.76 (0.88, 3.51) |  | 14 | 1.32 (0.69, 2.52) | 0.9508 |
| Kidney (renal cell carcinoma) | No obesity | 261 | 1.00 |  | 388 | 1.00 |  |
|  | Preclinical obesity | 121 | 1.54 (1.24, 1.92) |  | 185 | 1.42 (1.19, 1.70) | 0.9860 |
|  | Clinical obesity | 97 | 2.17 (1.70, 2.77) |  | 153 | 1.53 (1.26, 1.86) | 0.9179 |
| Liver (hepatocellular carcinoma) | No obesity | 60 | 1.00 |  | 83 | 1.00 |  |
|  | Preclinical obesity | 27 | 1.60 (1.00, 2.55) |  | 43 | 1.61 (1.11, 2.35) | 0.9867 |
|  | Clinical obesity | 22 | 1.87 (1.11, 3.13) |  | 80 | 3.52 (2.55, 4.87) | 0.9673 |
| Lung | No obesity | 723 | 1.00 |  | 1,534 | 1.00 |  |
|  | Preclinical obesity | 200 | 0.86 (0.73, 1.01) |  | 524 | 0.95 (0.86, 1.05) | 0.9179 |
|  | Clinical obesity | 183 | 1.04 (0.88, 1.23) |  | 609 | 1.21 (1.10, 1.34) | 0.3040 |
| Lymphoid leukemia | No obesity | 163 | 1.00 |  | 320 | 1.00 |  |
|  | Preclinical obesity | 44 | 0.89 (0.63, 1.24) |  | 87 | 0.83 (0.65, 1.05) | 0.6572 |
|  | Clinical obesity | 31 | 1.16 (0.78, 1.73) |  | 76 | 0.89 (0.69, 1.15) | 0.5625 |
| Malignant melanoma | No obesity | 907 | 1.00 |  | 985 | 1.00 |  |
|  | Preclinical obesity | 247 | 0.94 (0.81, 1.08) |  | 334 | 1.07 (0.94, 1.21) | 0.9179 |
|  | Clinical obesity | 115 | 0.88 (0.72, 1.08) |  | 229 | 1.00 (0.86, 1.16) | 0.6501 |
| Multiple myeloma | No obesity | 196 | 1.00 |  | 341 | 1.00 |  |
|  | Preclinical obesity | 65 | 1.14 (0.85, 1.51) |  | 121 | 1.11 (0.90, 1.37) | 0.6501 |
|  | Clinical obesity | 31 | 0.94 (0.64, 1.40) |  | 106 | 1.25 (0.99, 1.57) | 0.6501 |
| Myeloid leukemia | No obesity | 83 | 1.00 |  | 176 | 1.00 |  |
|  | Preclinical obesity | 33 | 1.29 (0.85, 1.96) |  | 53 | 0.89 (0.65, 1.22) | 0.9867 |
|  | Clinical obesity | 30 | 2.17 (1.40, 3.36) |  | 43 | 0.89 (0.63, 1.26) | 0.6501 |
| Non-Hodgkins lymphoma | No obesity | 333 | 1.00 |  | 530 | 1.00 |  |
|  | Preclinical obesity | 111 | 1.15 (0.92, 1.43) |  | 156 | 0.90 (0.75, 1.08) | 0.5625 |
|  | Clinical obesity | 47 | 0.86 (0.63, 1.18) |  | 137 | 1.02 (0.84, 1.24) | 0.1020 |
| Oesophagus (adenocarcinoma) | No obesity | 117 | 1.00 |  | 252 | 1.00 |  |
|  | Preclinical obesity | 62 | 1.69 (1.23, 2.32) |  | 114 | 1.42 (1.13, 1.78) | 0.6501 |
|  | Clinical obesity | 52 | 2.48 (1.76, 3.49) |  | 94 | 1.40 (1.10, 1.80) | 0.6501 |
| Oesoghagus (squamous cell carcinoma) | No obesity | 56 | 1.00 |  | 119 | 1.00 |  |
|  | Preclinical obesity | 12 | 0.70 (0.37, 1.31) |  | 19 | 0.49 (0.30, 0.80) | 0.6871 |
|  | Clinical obesity | 12 | 1.06 (0.55, 2.04) |  | 25 | 0.75 (0.48, 1.17) | 0.1470 |
| Ovary | No obesity | 269 | 1.00 |  | 296 | 1.00 |  |
|  | Preclinical obesity | 99 | 1.14 (0.90, 1.44) |  | 123 | 1.15 (0.92, 1.42) | 0.5625 |
|  | Clinical obesity | 57 | 1.10 (0.81, 1.48) |  | 88 | 1.08 (0.85, 1.39) | 0.7204 |
| Pancreas | No obesity | 219 | 1.00 |  | 483 | 1.00 |  |
|  | Preclinical obesity | 98 | 1.48 (1.16, 1.89) |  | 174 | 1.08 (0.90, 1.29) | 0.9179 |
|  | Clinical obesity | 68 | 1.78 (1.34, 2.37) |  | 170 | 1.34 (1.11, 1.61) | 0.9179 |
| Prostate (non-fatal) | No obesity | 2,418 | 1.00 |  | 4,881 | 1.00 |  |
|  | Preclinical obesity | 596 | 0.87 (0.79, 0.96) |  | 1,244 | 0.92 (0.86, 0.98) | 0.3360 |
|  | Clinical obesity | 279 | 0.82 (0.72, 0.93) |  | 914 | 0.89 (0.83, 0.96) | 0.5625 |
| Prostate (fatal) | No obesity | 101 | 1.00 |  | 395 | 1.00 |  |
|  | Preclinical obesity | 39 | 1.34 (0.91, 1.98) |  | 136 | 1.21 (0.99, 1.48) | 0.8828 |
|  | Clinical obesity | 22 | 1.37 (0.84, 2.24) |  | 95 | 1.12 (0.89, 1.42) | 0.6871 |
| Stomach (cardia) | No obesity | 42 | 1.00 |  | 95 | 1.00 |  |
|  | Preclinical obesity | 25 | 2.01 (1.21, 3.35) |  | 36 | 1.24 (0.84, 1.84) | 0.9179 |
|  | Clinical obesity | 16 | 2.21 (1.21, 4.04) |  | 39 | 1.59 (1.08, 2.35) | 0.6871 |
| Stomach (nnon-cardia) | No obesity | 33 | 1.00 |  | 79 | 1.00 |  |
|  | Preclinical obesity | 14 | 1.36 (0.71, 2.58) |  | 22 | 0.82 (0.51, 1.33) | 0.5625 |
|  | Clinical obesity | 4 | 0.68 (0.23, 1.97) |  | 21 | 0.83 (0.50, 1.37) | 0.6501 |
| Thyroid | No obesity | 143 | 1.00 |  | 91 | 1.00 |  |
|  | Preclinical obesity | 55 | 1.31 (0.96, 1.81) |  | 41 | 1.36 (0.93, 1.98) | 0.6501 |
|  | Clinical obesity | 47 | 1.86 (1.31, 2.63) |  | 26 | 1.17 (0.75, 1.85) | 0.9179 |
| Obesity-related | No obesity | 4,515 | 1.00 |  | 6,886 | 1.00 |  |
|  | Preclinical obesity | 1,869 | 1.34 (1.26, 1.41) |  | 3,050 | 1.27 (1.22, 1.33) | 0.9867 |
|  | Clinical obesity | 1,156 | 1.38 (1.29, 1.47) |  | 2,455 | 1.32 (1.26, 1.39) | 0.3360 |
| Overall | No obesity | 12,176 | 1.00 |  | 17,391 | 1.00 |  |
|  | Preclinical obesity | 3,966 | 1.09 (1.05, 1.13) |  | 6,165 | 1.09 (1.06, 1.12) | 0.6501 |
|  | Clinical obesity | 2,333 | 1.12 (1.07, 1.17) |  | 5,029 | 1.13 (1.10, 1.17) | 0.6501 |
| FDR: false discovery rate.  FDR was applied for each classification separately. | | | | | | | |

# Supplemental Table 5: Hazard ratios and 95% confidence intervals for preclinical obesity and clinical obesity in relation to smoking-related cancer types according to smoking status

| **Cancer type** | **Group** | **Never smoker** | |  | **Ever smoker** | | **FDR-adjusted**  **P-value for interaction** |
| --- | --- | --- | --- | --- | --- | --- | --- |
|  |  | **Cases** | **HR (95% CI)** |  | **Cases** | **HR (95% CI)** |  |
| Bladder | No obesity | 231 | 1.00 |  | 417 | 1.00 |  |
|  | Preclinical obesity | 72 | 1.15 (0.88, 1.52) |  | 170 | 1.16 (0.96, 1.39) | 0.9993 |
|  | Clinical obesity | 41 | 1.09 (0.77, 1.53) |  | 145 | 1.24 (1.02, 1.50) | 0.9993 |
| Head and neck | No obesity | 278 | 1.00 |  | 566 | 1.00 |  |
|  | Preclinical obesity | 83 | 1.14 (0.89, 1.46) |  | 174 | 0.90 (0.76, 1.07) | 0.9993 |
|  | Clinical obesity | 48 | 1.18 (0.86, 1.62) |  | 118 | 0.78 (0.63, 0.95) | 0.9993 |
| Kidney (renal cell carcinoma) | No obesity | 316 | 1.00 |  | 331 | 1.00 |  |
|  | Preclinical obesity | 136 | 1.55 (1.26, 1.90) |  | 166 | 1.38 (1.14, 1.67) | 0.9993 |
|  | Clinical obesity | 85 | 1.64 (1.28, 2.10) |  | 164 | 1.82 (1.50, 2.22) | 0.9993 |
| Liver (hepatocellular carcinoma) | No obesity | 48 | 1.00 |  | 95 | 1.00 |  |
|  | Preclinical obesity | 21 | 1.56 (0.92, 2.64) |  | 49 | 1.54 (1.08, 2.19) | 0.9993 |
|  | Clinical obesity | 24 | 2.52 (1.49, 4.26) |  | 77 | 3.00 (2.18, 4.12) | 0.9993 |
| Lung | No obesity | 331 | 1.00 |  | 1,904 | 1.00 |  |
|  | Preclinical obesity | 94 | 0.95 (0.75, 1.20) |  | 616 | 0.84 (0.76, 0.92) | 0.9993 |
|  | Clinical obesity | 84 | 1.26 (0.98, 1.62) |  | 700 | 1.06 (0.96, 1.15) | 0.9993 |
| Oesophageal (squamous cell carcinoma) | No obesity | 74 | 1.00 |  | 100 | 1.00 |  |
|  | Preclinical obesity | 12 | 0.51 (0.27, 0.94) |  | 19 | 0.55 (0.33, 0.90) | 0.9993 |
|  | Clinical obesity | 11 | 0.66 (0.35, 1.28) |  | 26 | 0.90 (0.58, 1.42) | 0.9993 |
| Pancreas | No obesity | 345 | 1.00 |  | 355 | 1.00 |  |
|  | Preclinical obesity | 117 | 1.18 (0.95, 1.46) |  | 153 | 1.18 (0.98, 1.44) | 0.9993 |
|  | Clinical obesity | 93 | 1.45 (1.14, 1.85) |  | 143 | 1.40 (1.14, 1.71) | 0.9993 |
| Stomach (cardia) | No obesity | 47 | 1.00 |  | 88 | 1.00 |  |
|  | Preclinical obesity | 17 | 1.36 (0.77, 2.41) |  | 44 | 1.52 (1.05, 2.21) | 0.9993 |
|  | Clinical obesity | 17 | 2.12 (1.18, 3.80) |  | 38 | 1.63 (1.10, 2.43) | 0.9993 |
| Smoking-related cancers combined | No obesity | 1,670 | 1.00 |  | 3,856 | 1.00 |  |
|  | Preclinical obesity | 552 | 1.17 (1.06, 1.30) |  | 1,391 | 0.98 (0.92, 1.04) | 0.9993 |
|  | Clinical obesity | 403 | 1.37 (1.22, 1.54) |  | 1,411 | 1.18 (1.11, 1.26) | 0.9993 |
| FDR: false discovery rate.  FDR was applied for each classification separately. | | | | | | | |

**Supplemental Table 6: Hazard ratios and 95% confidence intervals for preclinical obesity and clinical obesity in relation to cancer types after excluding the first two years of follow-up and underweight individuals, adjusting for ethnicity and family history of cancer, and handling missing covariate data using multiple imputation**

|  |  | **Excluding the first two**  **years of follow-up and**  **underweight individuals** | | **Adjusting for ethnicity and family history of cancer** | | | **Handling missing covariate data using multiple imputation** | | |
| --- | --- | --- | --- | --- | --- | --- | --- | --- | --- |
| **Cancer type** | **Group** | **Cases** | **HR (95% CI)** | **Cases** | | **HR (95% CI)** | **Cases** | | **HR (95% CI)** |
| Biliary tract | No obesity | 241 | 1.00 | 277 | 1.00 | | 277 | 1.00 | |
|  | Preclinical obesity | 109 | 1.33 (1.05, 1.68) | 121 | 1.29 (1.04, 1.61) | | 121 | 1.31 (1.05, 1.63) | |
|  | Clinical obesity | 105 | 1.66 (1.30, 2.11) | 120 | 1.68 (1.34, 2.11) | | 120 | 1.70 (1.36, 2.13) | |
| Bladder | No obesity | 547 | 1.00 | 651 | 1.00 | | 651 | 1.00 | |
|  | Preclinical obesity | 219 | 1.25 (1.07, 1.47) | 245 | 1.18 (1.01, 1.37) | | 245 | 1.18 (1.01, 1.37) | |
|  | Clinical obesity | 153 | 1.21 (1.01, 1.46) | 187 | 1.23 (1.04, 1.45) | | 187 | 1.22 (1.03, 1.45) | |
| Breast (post-menopausal) | No obesity | 3,070 | 1.00 | 3,709 | 1.00 | | 3,709 | 1.00 | |
|  | Preclinical obesity | 1,307 | 1.18 (1.11, 1.26) | 1,573 | 1.19 (1.12, 1.27) | | 1,573 | 1.19 (1.12, 1.27) | |
|  | Clinical obesity | 936 | 1.19 (1.10, 1.28) | 1,115 | 1.19 (1.10, 1.27) | | 1,115 | 1.18 (1.10, 1.27) | |
| Breast (pre-menopausal) | No obesity | 1,410 | 1.00 | 1,690 | 1.00 | | 1,690 | 1.00 | |
|  | Preclinical obesity | 418 | 1.05 (0.94, 1.18) | 470 | 1.00 (0.90, 1.11) | | 470 | 1.01 (0.91, 1.12) | |
|  | Clinical obesity | 225 | 0.99 (0.85, 1.15) | 270 | 1.01 (0.88, 1.16) | | 270 | 1.01 (0.89, 1.16) | |
| Cervix | No obesity | 61 | 1.00 | 77 | 1.00 | | 77 | 1.00 | |
|  | Preclinical obesity | 17 | 0.89 (0.51, 1.54) | 23 | 0.93 (0.57, 1.51) | | 23 | 0.91 (0.56, 1.47) | |
|  | Clinical obesity | 13 | 1.03 (0.55, 1.94) | 16 | 0.99 (0.56, 1.74) | | 16 | 0.96 (0.55, 1.69) | |
| Colorectum | No obesity | 2,949 | 1.00 | 3,457 | 1.00 | | 3,457 | 1.00 | |
|  | Preclinical obesity | 1,172 | 1.25 (1.16, 1.34) | 1,348 | 1.23 (1.15, 1.31) | | 1,348 | 1.23 (1.15, 1.31) | |
|  | Clinical obesity | 731 | 1.12 (1.03, 1.22) | 856 | 1.12 (1.04, 1.21) | | 856 | 1.12 (1.03, 1.21) | |
| Diffuse large B-cell lymphoma | No obesity | 420 | 1.00 | 470 | 1.00 | | 470 | 1.00 | |
|  | Preclinical obesity | 162 | 1.22 (1.02, 1.47) | 177 | 1.20 (1.01, 1.44) | | 177 | 1.21 (1.01, 1.45) | |
|  | Clinical obesity | 109 | 1.16 (0.93, 1.45) | 122 | 1.17 (0.95, 1.44) | | 122 | 1.18 (0.96, 1.46) | |
| Endometrial | No obesity | 525 | 1.00 | 622 | 1.00 | | 622 | 1.00 | |
|  | Preclinical obesity | 425 | 2.40 (2.10, 2.74) | 488 | 2.34 (2.07, 2.64) | | 488 | 2.35 (2.08, 2.66) | |
|  | Clinical obesity | 314 | 2.89 (2.49, 3.35) | 374 | 2.92 (2.55, 3.34) | | 374 | 2.92 (2.55, 3.35) | |
| Glioma | No obesity | 444 | 1.00 | 521 | 1.00 | | 521 | 1.00 | |
|  | Preclinical obesity | 118 | 0.85 (0.69, 1.05) | 134 | 0.82 (0.68, 1.00) | | 134 | 0.83 (0.68, 1.01) | |
|  | Clinical obesity | 75 | 0.84 (0.65, 1.08) | 91 | 0.85 (0.68, 1.07) | | 91 | 0.86 (0.68, 1.08) | |
| Head, neck, oral, pharynx, larynx | No obesity | 727 | 1.00 | 850 | 1.00 | | 850 | 1.00 | |
|  | Preclinical obesity | 230 | 1.03 (0.89, 1.20) | 259 | 1.00 (0.87, 1.16) | | 259 | 1.00 (0.87, 1.16) | |
|  | Clinical obesity | 145 | 0.93 (0.77, 1.12) | 167 | 0.91 (0.76, 1.08) | | 167 | 0.91 (0.77, 1.08) | |
| Hodgkins lymphoma | No obesity | 57 | 1.00 | 70 | 1.00 | | 70 | 1.00 | |
|  | Preclinical obesity | 30 | 1.43 (0.91, 2.26) | 33 | 1.35 (0.88, 2.07) | | 33 | 1.39 (0.91, 2.12) | |
|  | Clinical obesity | 22 | 1.47 (0.88, 2.47) | 26 | 1.52 (0.94, 2.44) | | 26 | 1.50 (0.93, 2.40) | |
| Kidney (renal cell carcinoma) | No obesity | 582 | 1.00 | 649 | 1.00 | | 649 | 1.00 | |
|  | Preclinical obesity | 270 | 1.44 (1.24, 1.67) | 306 | 1.47 (1.28, 1.69) | | 306 | 1.48 (1.29, 1.71) | |
|  | Clinical obesity | 219 | 1.72 (1.46, 2.03) | 250 | 1.76 (1.51, 2.05) | | 250 | 1.78 (1.53, 2.07) | |
| Liver (hepatocellular carcinoma) | No obesity | 131 | 1.00 | 143 | 1.00 | | 143 | 1.00 | |
|  | Preclinical obesity | 69 | 1.68 (1.25, 2.27) | 70 | 1.58 (1.18, 2.12) | | 70 | 1.60 (1.19, 2.15) | |
|  | Clinical obesity | 99 | 3.08 (2.34, 4.07) | 102 | 2.96 (2.26, 3.88) | | 102 | 3.01 (2.29, 3.94) | |
| Lung | No obesity | 1,967 | 1.00 | 2,257 | 1.00 | | 2,257 | 1.00 | |
|  | Preclinical obesity | 633 | 0.91 (0.83, 1.00) | 724 | 0.92 (0.85, 1.00) | | 724 | 0.93 (0.85, 1.01) | |
|  | Clinical obesity | 716 | 1.20 (1.10, 1.31) | 792 | 1.17 (1.07, 1.27) | | 792 | 1.18 (1.08, 1.28) | |
| Lymphoid leukemia | No obesity | 407 | 1.00 | 483 | 1.00 | | 483 | 1.00 | |
|  | Preclinical obesity | 117 | 0.90 (0.73, 1.12) | 131 | 0.85 (0.69, 1.03) | | 131 | 0.85 (0.70, 1.04) | |
|  | Clinical obesity | 92 | 0.98 (0.78, 1.25) | 107 | 0.96 (0.77, 1.20) | | 107 | 0.96 (0.77, 1.20) | |
| Malignant melanoma | No obesity | 1,615 | 1.00 | 1,892 | 1.00 | | 1,892 | 1.00 | |
|  | Preclinical obesity | 508 | 1.02 (0.92, 1.13) | 581 | 1.00 (0.91, 1.11) | | 581 | 1.00 (0.91, 1.10) | |
|  | Clinical obesity | 295 | 0.95 (0.84, 1.09) | 344 | 0.97 (0.86, 1.09) | | 344 | 0.95 (0.85, 1.07) | |
| Multiple myeloma | No obesity | 483 | 1.00 | 537 | 1.00 | | 537 | 1.00 | |
|  | Preclinical obesity | 171 | 1.14 (0.95, 1.36) | 186 | 1.12 (0.94, 1.33) | | 186 | 1.12 (0.95, 1.33) | |
|  | Clinical obesity | 118 | 1.11 (0.90, 1.37) | 137 | 1.15 (0.95, 1.40) | | 137 | 1.18 (0.97, 1.44) | |
| Myeloid leukemia | No obesity | 230 | 1.00 | 259 | 1.00 | | 259 | 1.00 | |
|  | Preclinical obesity | 81 | 1.08 (0.83, 1.40) | 86 | 1.02 (0.80, 1.32) | | 86 | 1.04 (0.81, 1.33) | |
|  | Clinical obesity | 64 | 1.16 (0.87, 1.56) | 73 | 1.20 (0.91, 1.57) | | 73 | 1.20 (0.92, 1.58) | |
| Non-Hodgkins lymphoma | No obesity | 736 | 1.00 | 863 | 1.00 | | 863 | 1.00 | |
|  | Preclinical obesity | 238 | 1.02 (0.88, 1.19) | 267 | 0.99 (0.86, 1.14) | | 267 | 0.99 (0.86, 1.14) | |
|  | Clinical obesity | 147 | 0.91 (0.76, 1.10) | 184 | 0.98 (0.83, 1.16) | | 184 | 0.98 (0.83, 1.15) | |
| Oesophagus (adenocarcinoma) | No obesity | 315 | 1.00 | 369 | 1.00 | | 369 | 1.00 | |
|  | Preclinical obesity | 165 | 1.64 (1.35, 1.99) | 176 | 1.49 (1.24, 1.79) | | 176 | 1.50 (1.24, 1.80) | |
|  | Clinical obesity | 125 | 1.67 (1.35, 2.08) | 146 | 1.67 (1.37, 2.04) | | 146 | 1.68 (1.37, 2.05) | |
| Oesoghagus (squamous cell carcinoma) | No obesity | 149 | 1.00 | 175 | 1.00 | | 175 | 1.00 | |
|  | Preclinical obesity | 27 | 0.56 (0.37, 0.85) | 31 | 0.55 (0.37, 0.81) | | 31 | 0.55 (0.38, 0.82) | |
|  | Clinical obesity | 34 | 0.94 (0.64, 1.38) | 37 | 0.85 (0.59, 1.23) | | 37 | 0.85 (0.59, 1.23) | |
| Ovary | No obesity | 468 | 1.00 | 565 | 1.00 | | 565 | 1.00 | |
|  | Preclinical obesity | 195 | 1.20 (1.01, 1.43) | 222 | 1.15 (0.98, 1.35) | | 222 | 1.15 (0.98, 1.35) | |
|  | Clinical obesity | 122 | 1.09 (0.88, 1.34) | 145 | 1.10 (0.91, 1.33) | | 145 | 1.09 (0.90, 1.32) | |
| Pancreas | No obesity | 630 | 1.00 | 702 | 1.00 | | 702 | 1.00 | |
|  | Preclinical obesity | 248 | 1.22 (1.05, 1.41) | 272 | 1.20 (1.04, 1.38) | | 272 | 1.20 (1.04, 1.38) | |
|  | Clinical obesity | 210 | 1.43 (1.21, 1.68) | 238 | 1.45 (1.24, 1.69) | | 238 | 1.45 (1.24, 1.69) | |
| Prostate (non-fatal) | No obesity | 6,404 | 1.00 | 7,299 | 1.00 | | 7,299 | 1.00 | |
|  | Preclinical obesity | 1,633 | 0.91 (0.86, 0.97) | 1,840 | 0.91 (0.86, 0.96) | | 1,840 | 0.90 (0.86, 0.95) | |
|  | Clinical obesity | 1,025 | 0.87 (0.81, 0.93) | 1,193 | 0.88 (0.82, 0.94) | | 1,193 | 0.87 (0.82, 0.93) | |
| Prostate (fatal) | No obesity | 485 | 1.00 | 496 | 1.00 | | 496 | 1.00 | |
|  | Preclinical obesity | 174 | 1.30 (1.08, 1.56) | 175 | 1.27 (1.06, 1.52) | | 175 | 1.27 (1.06, 1.52) | |
|  | Clinical obesity | 113 | 1.21 (0.97, 1.50) | 117 | 1.21 (0.98, 1.50) | | 117 | 1.21 (0.98, 1.50) | |
| Stomach (cardia) | No obesity | 116 | 1.00 | 137 | 1.00 | | 137 | 1.00 | |
|  | Preclinical obesity | 56 | 1.61 (1.16, 2.23) | 61 | 1.46 (1.07, 1.99) | | 61 | 1.46 (1.07, 2.00) | |
|  | Clinical obesity | 49 | 1.92 (1.35, 2.73) | 55 | 1.77 (1.27, 2.46) | | 55 | 1.77 (1.27, 2.46) | |
| Stomach (non-cardia) | No obesity | 94 | 1.00 | 112 | 1.00 | | 112 | 1.00 | |
|  | Preclinical obesity | 34 | 1.09 (0.73, 1.62) | 36 | 0.98 (0.67, 1.44) | | 36 | 0.97 (0.66, 1.42) | |
|  | Clinical obesity | 21 | 0.82 (0.50, 1.34) | 25 | 0.82 (0.52, 1.28) | | 25 | 0.80 (0.51, 1.26) | |
| Thyroid | No obesity | 192 | 1.00 | 234 | 1.00 | | 234 | 1.00 | |
|  | Preclinical obesity | 85 | 1.42 (1.09, 1.84) | 96 | 1.34 (1.05, 1.71) | | 96 | 1.35 (1.06, 1.72) | |
|  | Clinical obesity | 63 | 1.59 (1.18, 2.15) | 73 | 1.55 (1.18, 2.05) | | 73 | 1.57 (1.19, 2.07) | |
| Obesity-related | No obesity | 9,702 | 1.00 | 11,401 | 1.00 | | 11,401 | 1.00 | |
|  | Preclinical obesity | 4,272 | 1.31 (1.26, 1.36) | 4,919 | 1.29 (1.25, 1.34) | | 4,919 | 1.29 (1.25, 1.34) | |
|  | Clinical obesity | 3,091 | 1.34 (1.28, 1.40) | 3,611 | 1.34 (1.29, 1.39) | | 3,611 | 1.34 (1.29, 1.39) | |
| Overall | No obesity | 25,456 | 1.00 | 29,567 | 1.00 | | 29,567 | 1.00 | |
|  | Preclinical obesity | 8,911 | 1.11 (1.08, 1.14) | 10,131 | 1.09 (1.06, 1.11) | | 10,131 | 1.09 (1.07, 1.12) | |
|  | Clinical obesity | 6,340 | 1.13 (1.10, 1.16) | 7,362 | 1.13 (1.10, 1.16) | | 7,362 | 1.13 (1.10, 1.16) | |

**Supplemental Table 7: Hazard ratios and 95% confidence intervals for preclinical obesity and clinical obesity in relation**

**to cancer types after restricting analyses to ICD-based organ dysfunction indicators**

| **Cancer type** | **Group** | **Cases** | **HR (95% CI)** |
| --- | --- | --- | --- |
| Biliary tract | No obesity | 277 | 1.00 |
|  | Preclinical obesity | 189 | 1.40 (1.16, 1.70) |
|  | Clinical obesity | 52 | 1.69 (1.24, 2.30) |
| Bladder | No obesity | 651 | 1.00 |
|  | Preclinical obesity | 340 | 1.18 (1.03, 1.35) |
|  | Clinical obesity | 92 | 1.26 (1.01, 1.58) |
| Breast (post-menopausal) | No obesity | 3,709 | 1.00 |
|  | Preclinical obesity | 2,266 | 1.17 (1.11, 1.24) |
|  | Clinical obesity | 422 | 1.28 (1.16, 1.42) |
| Breast (pre-menopausal) | No obesity | 1,690 | 1.00 |
|  | Preclinical obesity | 689 | 0.99 (0.91, 1.09) |
|  | Clinical obesity | 51 | 1.09 (0.82, 1.46) |
| Cervix | No obesity | 77 | 1.00 |
|  | Preclinical obesity | 34 | 0.95 (0.62, 1.44) |
|  | Clinical obesity | 5 | 1.08 (0.42, 2.75) |
| Colorectum | No obesity | 3,457 | 1.00 |
|  | Preclinical obesity | 1,799 | 1.18 (1.11, 1.25) |
|  | Clinical obesity | 405 | 1.23 (1.11, 1.37) |
| Diffuse large B-cell lymphoma | No obesity | 470 | 1.00 |
|  | Preclinical obesity | 241 | 1.17 (1.00, 1.38) |
|  | Clinical obesity | 58 | 1.27 (0.96, 1.68) |
| Endometrial | No obesity | 622 | 1.00 |
|  | Preclinical obesity | 727 | 2.44 (2.18, 2.73) |
|  | Clinical obesity | 135 | 3.40 (2.80, 4.13) |
| Glioma | No obesity | 521 | 1.00 |
|  | Preclinical obesity | 189 | 0.84 (0.70, 0.99) |
|  | Clinical obesity | 36 | 0.82 (0.58, 1.15) |
| Head, neck, oral, pharynx, larynx | No obesity | 850 | 1.00 |
|  | Preclinical obesity | 343 | 0.94 (0.83, 1.07) |
|  | Clinical obesity | 83 | 1.09 (0.86, 1.37) |
| Hodgkins lymphoma | No obesity | 70 | 1.00 |
|  | Preclinical obesity | 50 | 1.43 (0.98, 2.09) |
|  | Clinical obesity | 9 | 1.29 (0.63, 2.64) |
| Kidney (renal cell carcinoma) | No obesity | 649 | 1.00 |
|  | Preclinical obesity | 435 | 1.50 (1.33, 1.71) |
|  | Clinical obesity | 121 | 1.97 (1.61, 2.41) |
| Liver (hepatocellular carcinoma) | No obesity | 143 | 1.00 |
|  | Preclinical obesity | 118 | 1.88 (1.46, 2.42) |
|  | Clinical obesity | 54 | 3.28 (2.36, 4.56) |
| Lung | No obesity | 2,257 | 1.00 |
|  | Preclinical obesity | 1,159 | 0.98 (0.91, 1.05) |
|  | Clinical obesity | 357 | 1.26 (1.12, 1.41) |
| Lymphoid leukemia | No obesity | 483 | 1.00 |
|  | Preclinical obesity | 182 | 0.84 (0.70, 1.00) |
|  | Clinical obesity | 56 | 1.14 (0.86, 1.52) |
| Melanoma | No obesity | 1,892 | 1.00 |
|  | Preclinical obesity | 771 | 0.97 (0.89, 1.06) |
|  | Clinical obesity | 154 | 1.07 (0.90, 1.26) |
| Multiple myeloma | No obesity | 537 | 1.00 |
|  | Preclinical obesity | 251 | 1.08 (0.93, 1.26) |
|  | Clinical obesity | 72 | 1.44 (1.12, 1.86) |
| Myeloid leukemia | No obesity | 259 | 1.00 |
|  | Preclinical obesity | 131 | 1.10 (0.89, 1.37) |
|  | Clinical obesity | 28 | 1.05 (0.71, 1.57) |
| Non-Hodgkins lymphoma | No obesity | 863 | 1.00 |
|  | Preclinical obesity | 377 | 1.00 (0.88, 1.13) |
|  | Clinical obesity | 74 | 0.92 (0.72, 1.18) |
| Oesophagus (adenocarcinoma) | No obesity | 369 | 1.00 |
|  | Preclinical obesity | 263 | 1.60 (1.36, 1.89) |
|  | Clinical obesity | 59 | 1.41 (1.06, 1.86) |
| Oesoghagus (squamous cell carcinoma) | No obesity | 175 | 1.00 |
|  | Preclinical obesity | 50 | 0.61 (0.44, 0.84) |
|  | Clinical obesity | 18 | 0.99 (0.60, 1.63) |
| Ovary | No obesity | 565 | 1.00 |
|  | Preclinical obesity | 319 | 1.13 (0.98, 1.30) |
|  | Clinical obesity | 48 | 1.11 (0.82, 1.50) |
| Pancreas | No obesity | 702 | 1.00 |
|  | Preclinical obesity | 402 | 1.25 (1.10, 1.42) |
|  | Clinical obesity | 108 | 1.54 (1.25, 1.89) |
| Prostate (non-fatal) | No obesity | 7,299 | 1.00 |
|  | Preclinical obesity | 2,422 | 0.89 (0.85, 0.94) |
|  | Clinical obesity | 611 | 0.89 (0.82, 0.97) |
| Prostate (fatal) | No obesity | 496 | 1.00 |
|  | Preclinical obesity | 236 | 1.28 (1.08, 1.51) |
|  | Clinical obesity | 56 | 1.12 (0.84, 1.49) |
| Stomach (cardia) | No obesity | 137 | 1.00 |
|  | Preclinical obesity | 95 | 1.63 (1.24, 2.13) |
|  | Clinical obesity | 21 | 1.44 (0.90, 2.30) |
| Stomach (non-cardia) | No obesity | 112 | 1.00 |
|  | Preclinical obesity | 50 | 0.94 (0.67, 1.33) |
|  | Clinical obesity | 11 | 0.79 (0.42, 1.49) |
| Thyroid | No obesity | 234 | 1.00 |
|  | Preclinical obesity | 144 | 1.39 (1.12, 1.73) |
|  | Clinical obesity | 25 | 1.56 (1.02, 2.40) |
| Obesity-related | No obesity | 11,401 | 1.00 |
|  | Preclinical obesity | 7,008 | 1.29 (1.25, 1.33) |
|  | Clinical obesity | 1,522 | 1.44 (1.36, 1.52) |
| Overall | No obesity | 29,567 | 1.00 |
|  | Preclinical obesity | 14,272 | 1.09 (1.07, 1.11) |
|  | Clinical obesity | 3,221 | 1.19 (1.14, 1.23) |
